# Supplementary material for: Nuclear Pore-Like Structures in a Compartmentalized Bacterium
Source: PLoS One. 2017 Feb 1;12(2):e0169432. doi: 10.1371/journal.pone.0169432 (PMC5287468; doi:10.1371/journal.pone.0169432)
Supplement: S1 Text — (DOCX) [file pone.0169432.s001.docx]

**S Text**

**Bioinformatics analyses**

We performed a number of bioinformatics analyses on the 128 unique proteins identified through proteomics as belonging to the pore-containing membrane fraction (fraction 3). We first searched for similarity to known proteins in the non-redundant protein database (downloaded from NCBI) using BLASTP and PHMMER.

BLASTP (Altschul et al. 1997) reported 112 proteins with significant homologs (E<0.001) (S3 Table). Of these, 33 are in the membrane fraction 3 (84.6% of all fraction 3 proteins). Of the significant hits, 25 top hits are to non-identical sequences in *G. obscuriglobus*, 18 are to *Planctomyces*, and 16 to *S. acidiphila*. One sequence found no hits at all (ZP_02735547). Almost half of all hits are to hypothetical (50), unnamed (5) or probable (2) proteins. Among hits with assigned function, 6 are flagellar, 12 ribosomal, 6 are membrane-related, and 4 are efflux proteins.

PHMMER (from the HMMER package, ver. 3.0([1](#_ENREF_1))), returned significant hits for 115 proteins (E<0.001) (S3 Table), of which 21 are to *G. obscuriglobus*, 20 are to *Planctomyces*, and 21 are to *S. acidiphila*. The functional distribution is similar to the BLAST results: A large fraction are hypothetical (51), unnamed (7) and probable (3), while annotated functions include flagellar (6), ribosomal (13), efflux (4) and membrane (5) hits. Notably, PHMMER and BLASTP find the exact same top hits for 64/128 proteins. 17 are non-identical *G. obscuriglobus* proteins, 11 are from *Planctomyces*, and 14 from *S. acidiphila*.

As BLASTP and PHMMER screens both identified hits to other proteins coded in the *G. obscuriglobus* genome*,* we examined similarity among proteins from the pore-containing fraction. We performed all against all BLAST ([2](#_ENREF_2)) followed by a Markov clustering on the results ([3](#_ENREF_3)) as implemented in VisBLAST ([4](#_ENREF_4)) with default parameters E<0.001 and i-value=2.0. This yielded two large clusters (both containing 11 proteins), and a number of small triplet and doublet clusters (Fig S13). The vast majority of proteins were singletons (91 proteins; S3 Table), and the clustering was robust at higher E-value cutoffs (up to E=10) and i-values (from 1.2 to 5). One of these large clusters contained 7 proteins that were unique to fraction 3 with the remaining four proteins being divided between fractions (2,3) and (2,3,6). The second large cluster contained no proteins that were unique to fraction 3. We performed the same clustering on the full set of all 512 proteins and the same clusters were recreated. VisBLAST was used to cluster proteins based on sequence similarity. An E-value cut-off of 0.001 was used together with an i-value of 2. Interestingly, the cluster containing 8 proteins unique to fraction 3 did not change at all in this larger analysis, which indicates that it is indeed membrane-specific. The other large cluster expanded with proteins belonging to fractions (2,6), (6) and especially fraction (2). Other large clusters were found within the set of 512 proteins, however none contained fraction 3 proteins (Fig S14).

Transmembrane helix structure potential was predicted for all 128 fraction 3 sequences using TMHMM2 ([5](#_ENREF_5)). Of these, 42 showed significant signal of one or more transmembrane helices. Seven of these are unique to fraction 3 (S3 Table). We also looked for evidence of coiled coils using Paircoil2 ([6](#_ENREF_6)). We report 15 proteins with significant signal of a coiled coil structure (S3 Table).

We next examined fold architecture of proteins in the membrane pore fraction using Phyre2 ([7](#_ENREF_7)). This approach uses homology modelling to infer the structure of an amino acid sequence based on resemblance to known structures. 127 of the sequences were modelled in full, but one protein (ZP_02734818, 2558 aa) was analyzed in pieces because of its large size. The Phyre2 result for this protein is thus based on the best scoring subsequence. In the S3 Table we report the top Phyre2 structural hit for each protein. These hits are extracted automatically from the output, and we note that they may not represent the sole best hit (multiple hits often have the same highest confidence score). Based on PDB descriptions, these unfiltered results include membrane proteins (14), flagellar proteins (7) and ribosomal proteins (8), along with a number of other bacterial membrane/transport-related hits.

Comparing all structural predictions with our clustering analysis reveals a number of interesting patterns. Most significantly, cluster 1 consists of proteins modelled by Phyre2 as β-propeller-containing. This is notable given the presence of the β-propeller architecture in protein constituents of the eukaryotic nuclear pore complex ([8](#_ENREF_8)). Of the 11 proteins in this β-propeller cluster, seven are unique to the pore-containing membrane fraction (3) (Fig S14 and S3 Table). The second large cluster (cluster 2) is dominated by pilins, and the proteins in this cluster mainly come from membrane fraction (3, 2, 6). Approximately half the structural predictions for singleton proteins showed significant structural similarity to porins and membrane proteins, ribosomal subunits, and flagellar proteins (S3 Table). The predicted triplet cluster contains flagellar proteins.

A closer investigation of the structure predictions for the 11 members of the cluster 1 showed that eight yield at least one structure prediction with a confidence >95%. In most cases, multiple predictions are made covering all parts of the sequences.

As constituency in a cluster does not establish whether all constituents share a common region of sequence similarity, we performed multiple sequence alignments across all members of both cluster 1 and cluster 2 (using MAFFT, option L-ins-i, ([9](#_ENREF_9)). For cluster 1, all sequences displayed similarity in the C-terminal region. Fig S15 shows the alignment for the eight sequences for which we also obtained high confidence (>95%) structures with Phyre. We evaluated the full alignment using the T-Coffee CORE program ([10](#_ENREF_10)), which shows a moderately robust 8-way alignment with a CORE-score of 69 (where 100 is perfect alignment). It is clear that the conservation is most pronounced in the C-terminal end of the sequences from around position 850 in the alignment. Indeed, if only the C-terminal part of the alignment is analysed using T-Coffee, the score increases to 81. This corresponds well with the observation that the majority of hits retrieved when searching the non-redundant protein database using both BLAST and PHMMER are also against the C-terminal ends.

With the aim of better characterising the commonalities of cluster 1, we focused on the structural predictions associated with the common C-terminal region. Note that there is some disagreement between the top hits shown in S3 Table (which was automatically generated) and those derived from the conserved C-terminal region (S4 Table). If both coverage and confidence scores generated by Phyre 2 are considered, the structures associated with the C-terminal region (S4 Table) emerge as the best hits. Some cluster 1 proteins also yield significant predictions for their N-terminal ends, but these are not in conflict with results from their respective C-termini, indicating these may be multi-domain proteins. In all 8 cases where a significant (confidence >95%) structure model is obtained, the C-terminal predictions are for β-propeller structures that overlap with the conserved C-terminal region of the sequences (Fig S15). Furthermore, Phyre2 modeled all 8 proteins to the same PDB template (2C4D), which we interpret as independent verification that these proteins share a common structural fold. These results are not due to extensive sequence similarity, as the overall sequence identity between the queries and the PDB template ranges from 13% to 19%.

Results from our structural analysis of cluster 1 are given in S4 Table, and structural models are depicted in Fig 7C (all structures are visualized using The PyMOL Molecular Graphics System, Version 1.4.1 Schrödinger, LLC).

For the second large cluster (Fig S14), we performed the same type of analysis. All can be aligned (Fig S16), 10 of 11 sequences have significant (>95% confidence) structure predictions, and in all cases the best hit in terms of both confidence and coverage (S5 Table) was modeled against PDB file 1OQW. The predicted structures are all very similar and consist of a single α-helix.

Two proteins (ZP_02735673 and ZP_02736511) show possible α-solenoid structures with stacked α-helices (Fig S18). Both are singletons in the cluster analysis, and both are present in membrane fraction 3 (the first is in both fractions 3 and 2, and the latter is unique to fraction 3). The left-hand structure in Fig S18 (ZP_02735673) models against alpha-solenoid structures in pdb with high (>99%) confidence, with models spanning >95% of the sequence. The model shown is based on 1OYZ, a hypothetical protein from *E. coli*, which is classified in SCOP as a member of the ARM repeat superfamily. Within the top 10 hits are structures that derive from Bacteria, Archaea and Eukaryotes, including clathrin adaptor core proteins (2VGL, 1W63). The right-hand structure in Fig S18 (ZP_02736511) contains two high confidence domain models. The N-terminal region models to the same alpha-solenoid structure as seen in the left-hand structure in Fig S18. This spans 30% of the protein sequence. In the adjacent central region, Phyre2 models a response regulator (top hit: 1ZES) with high (>99%) confidence.

A possible FG repeat-containing protein (ZP_02734840) was found in the pore-containing membrane fraction and in the total nuclear membrane proteome. With 5 FGs in the first 200 residues, this conforms to a recent definition of FG-repeat nucleoporin ([11](#_ENREF_11)) but the C-terminal half of the protein models as a transmembrane beta-barrel protein.

We also performed a more general screen for bacterial transmembrane proteins among our structural predictions. To do this, we screened results for predicted structures of the following type: “outer membrane lipoprotein”, “outer membrane efflux protein”, “transmembrane beta barrel”, “porin”, “tolc” and “oprd/g/h/p/”. The same protein might have hits in more than one of these categories. For transmembrane beta barrels, we chose the best hit to a beta barrel spanning the membrane even if the term “transmembrane” was not used to describe that particular hit (however, for all proteins in this category at least one hit is called “transmembrane”). For all other categories we chose the best hit containing the specified keyword(s). S6 Table summarises these hits with their ID, membrane fraction, confidence score and coverage (as reported by Phyre2), and the PDB template used. For the oprx group we also list the specific class.

**Supplementary Text References**

1. Finn RD, Clements J, Eddy SR (2011) HMMER web server: interactive sequence similarity searching. *Nucleic acids research* **39**(Web Server issue):W29-37.

2. Altschul SF*,* et al. (1997) Gapped BLAST and PSI-BLAST: a new generation of protein database search programs. *Nucleic acids research* **25**(17):3389-3402.

3. van Dongen S (2000) Graph clustering by flow simulation. . PhD (University of Utrecht).

4. Weirather JL, Wilson ME, Donelson JE (2012) Mapping of VSG similarities in *Trypanosoma brucei*. *Molecular and biochemical parasitology* **181**(2):141-152.

5. Krogh A, Larsson B, von Heijne G, Sonnhammer EL (2001) Predicting transmembrane protein topology with a hidden Markov model: application to complete genomes. *Journal of molecular biology* **305**(3):567-580.

6. McDonnell AV, Jiang T, Keating AE, Berger B (2006) Paircoil2: improved prediction of coiled coils from sequence. *Bioinformatics* **22**(3):356-358.

7. Kelley LA, Sternberg MJE (2009) Protein structure prediction on the Web: a case study using the Phyre server. *Nat Protoc* **4**(3):363-371.

8. Devos D*,* et al. (2004) Components of coated vesicles and nuclear pore complexes share a common molecular architecture. *PLoS Biol* **2**(12):e380.

9. Katoh K, Toh H (2010) Parallelization of the MAFFT multiple sequence alignment program. *Bioinformatics* **26**(15):1899-1900.

10. Notredame C, Higgins DG, Heringa J (2000) T-Coffee: A novel method for fast and accurate multiple sequence alignment. *Journal of molecular biology* **302**(1):205-217.

11. Degrasse JA, Devos D (2010) A functional proteomic study of the *Trypanosoma brucei* nuclear pore complex: an informatic strategy. *Methods Mol Biol* **673**:231-238.
