# Supplementary figures and images for: Nuclear Pore-Like Structures in a Compartmentalized Bacterium
Source: PLoS One. 2017 Feb 1;12(2):e0169432. doi: 10.1371/journal.pone.0169432 (PMC5287468; doi:10.1371/journal.pone.0169432)

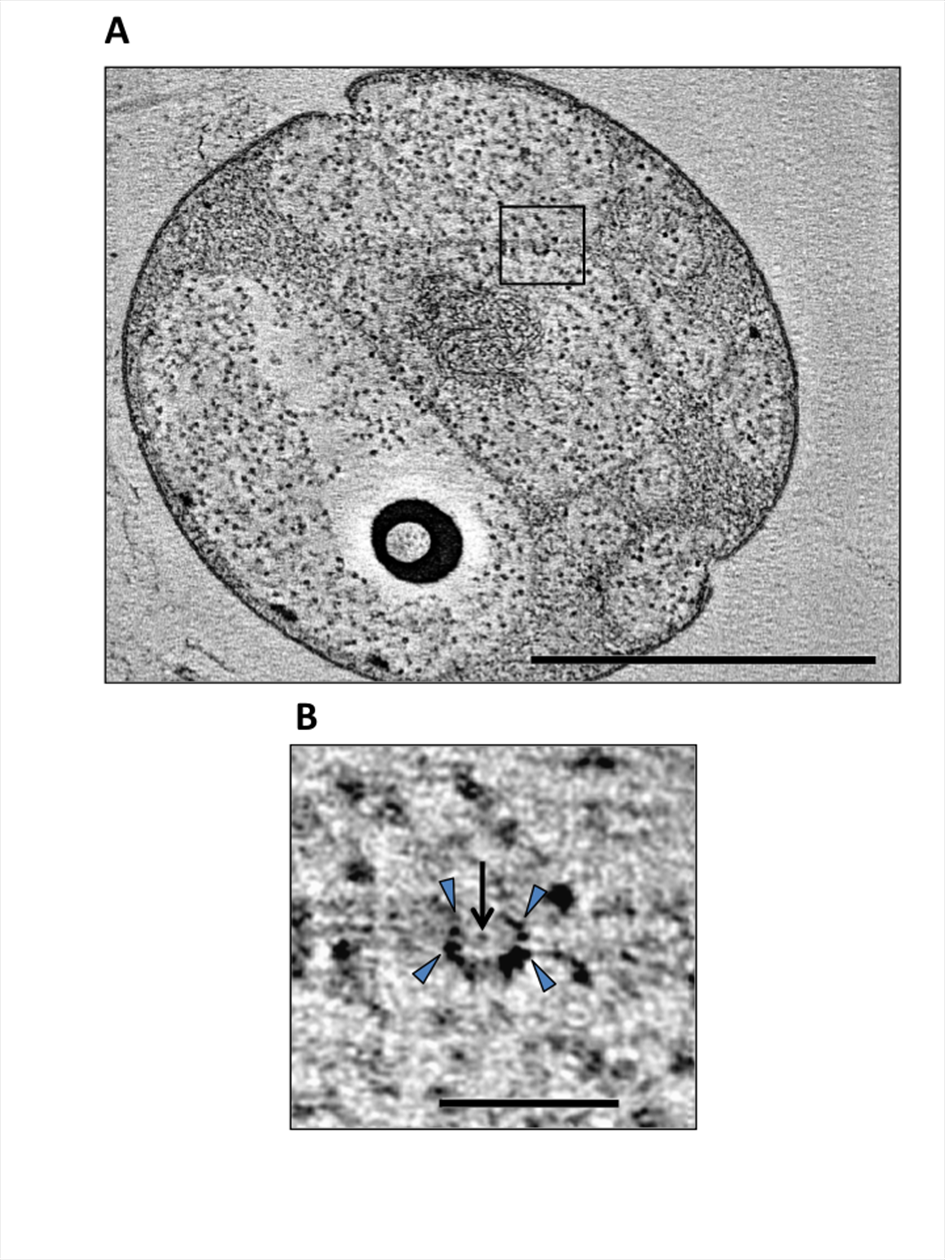

Supplement: S1 Fig — (A)Transmission electron micrograph of a tomographic slice of high-pressure-frozen cryosubstituted thick-sectioned cell showing a pore (boxed region) embedded in internal membranes situated within the cytoplasm and bounding the nuclear body region containing the cell’s nucleoid. Bar, 1 μm. Inset: (B) Enlarged view of the pore outlined by the box in (A)–the circular pore (blue arrowheads) is seen tilted en face and displays an inner and outer ring structure and a relatively electron-dense central plug (black arrow). Bar, 100 nm. (TIF) [file pone.0169432.s005.tif]

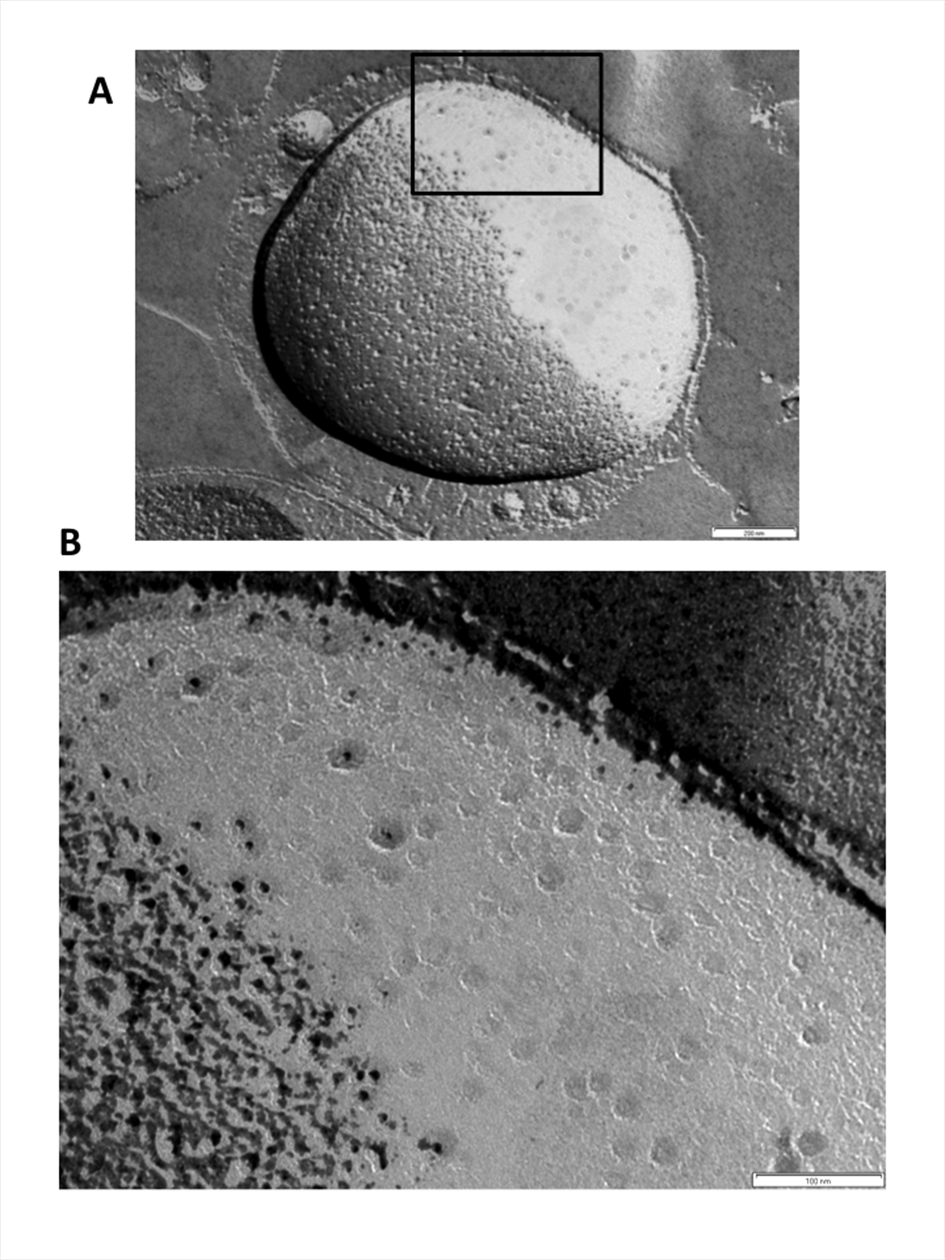

Supplement: S2 Fig — (A) Transmission electron micrograph of a platinum/carbon (Pt/C)-shadowed replica of a whole cell of Gemmata CJuql4 (ACM5157) which has been prepared via the freeze-fracture technique. The fractured whole cell contains a large spherical organelle taking up most of the cell volume, the surface of which has been fractured along a membrane. Pores are visible on the fractured membrane surface of this major cell compartment, interpreted as the nuclear body (e.g. in the boxed region) Bar, 200 nm. (B) An enlarged view of the boxed region of the freeze-fractured cell seen in (A) showing a region of a membrane surface where pore structures are visible. Several pores display substructure consistent with complex structure including a dark central core and a lighter ring surrounding the core (arrows). Other circular structures in the same size range are also visible but do not present this complex core-ring structure as clearly, presumably reflecting angle at which Pt/C metal shadow has been deposited during formation of the replica after fracture of the frozen cell. Bar, 100 nm. (TIF) [file pone.0169432.s006.tif]

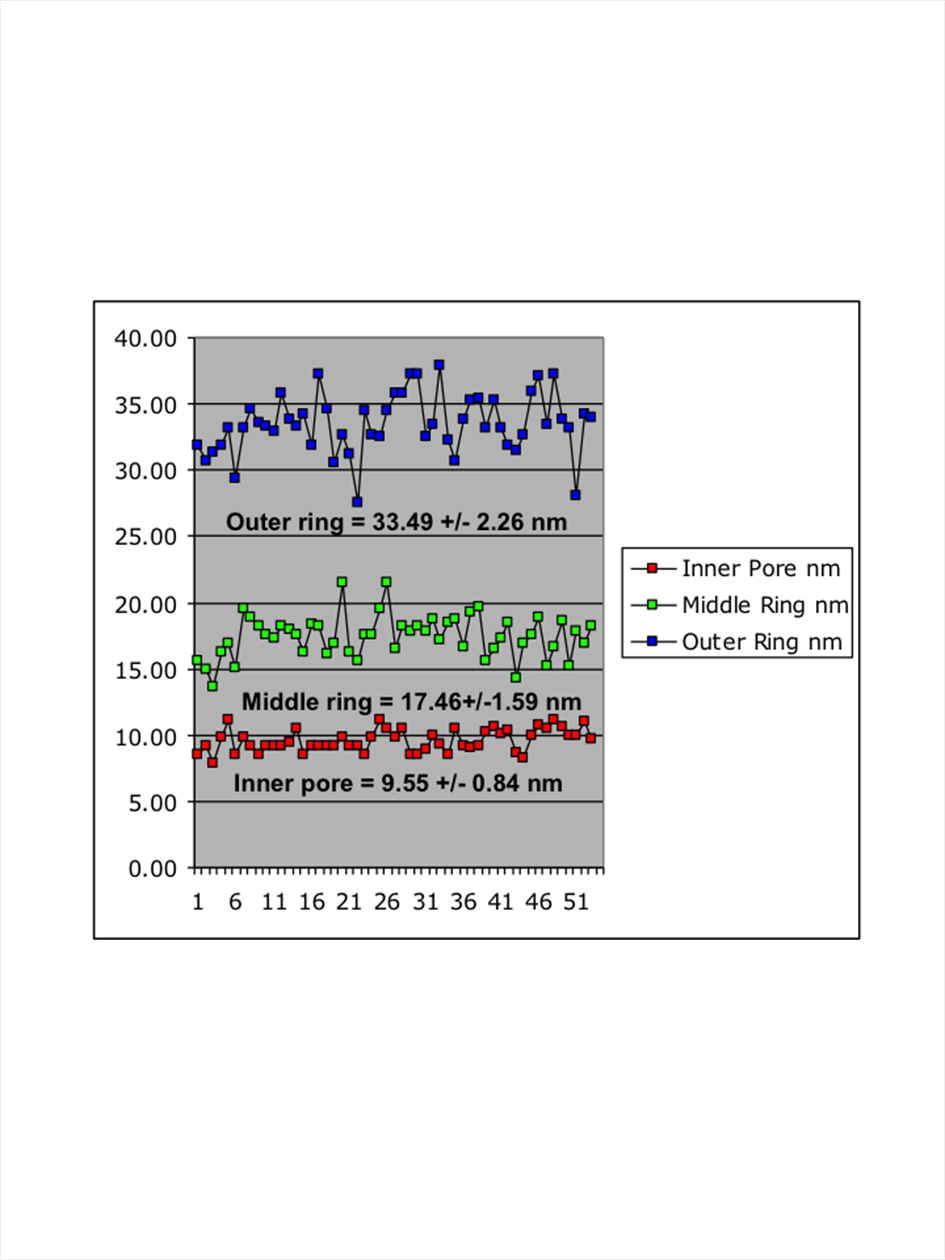

Supplement: S3 Fig — The dimensions were calculated from transmission electron micrographs of the membrane fragments released from lysed cells via sonication and negatively stained with ammonium molybdate. The pores usually appear as circular structures with dense pore centers surrounded by a thin lighter inner ring and a thicker outer ring (see Fig 3 for example). The bars are generated automatically and calculated by microscope software. (TIF) [file pone.0169432.s007.tif]

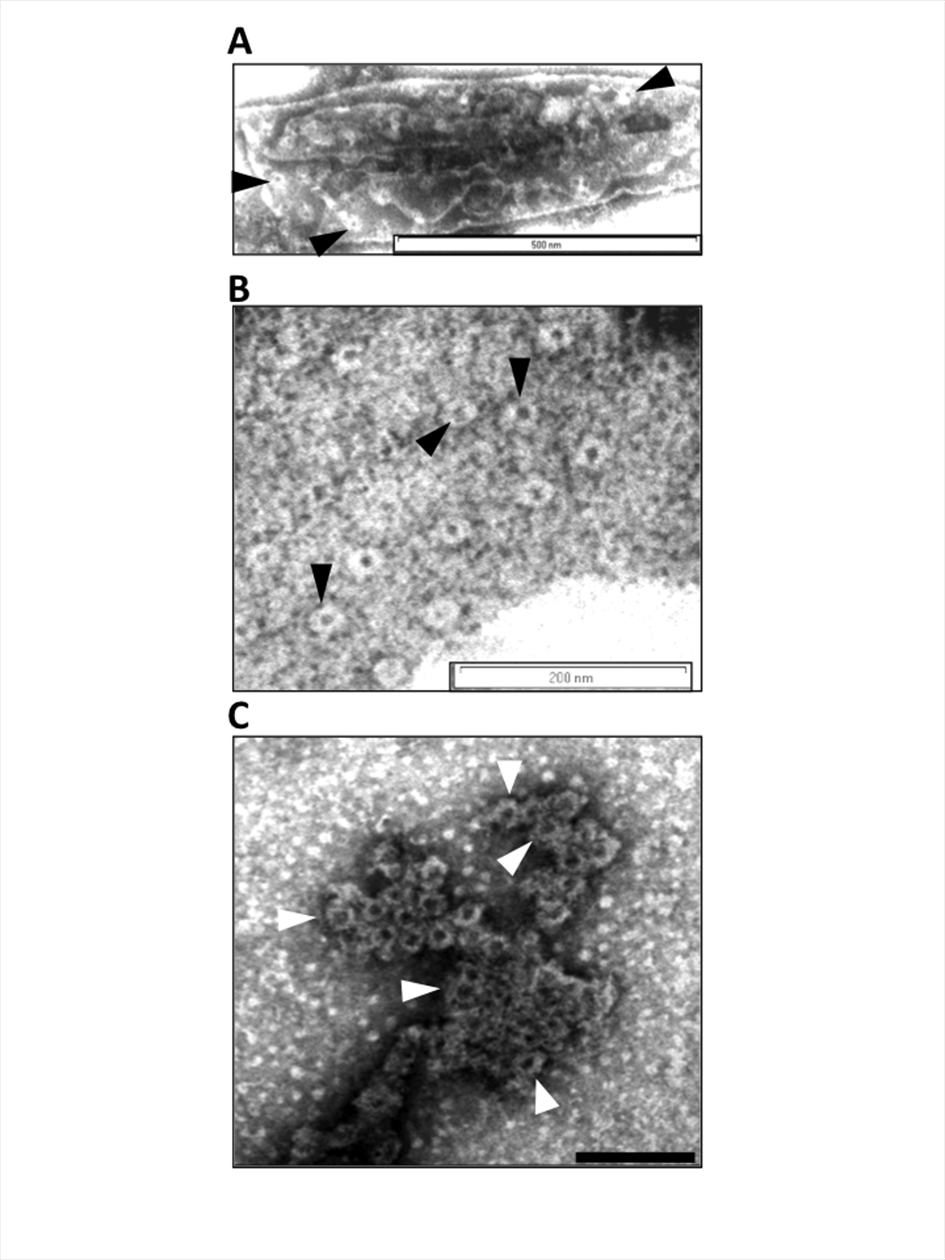

Supplement: S4 Fig — (A) Transmission electron micrograph of negatively stained gradient-fractionated pore-containing membranes purified from sonicated G. obscuriglobus acting as control for detergent treatments shown in (B) and (C). A “canoe” structure with pores (arrowheads) is visible. Bar, 500 nm. (B) Transmission electron micrograph of negatively stained gradient-fractionated pore-containing membranes purified from sonicated G. obscuriglobus after treatment with 1% Triton X-100 and 1% sodium deoxycholate detergent for 5 min. Pores (arrowheads) are visible within a partially degraded membrane background. Bar, 200 nm. (C) Transmission electron micrograph of negatively stained aggregated pore complexes seen after treatment of gradient-fractionated pore-containing membranes with 1% Triton X-100 and 1% sodium deoxycholate detergent for 30 min. Individual pores show a central dense core surrounded by a light ring and in some cases material projecting from the outer rim of the ring possibly representing spokes normally connecting inner to outer ring in intact pore complexes (arrowheads). Bar, 50 nm. (TIF) [file pone.0169432.s008.tif]

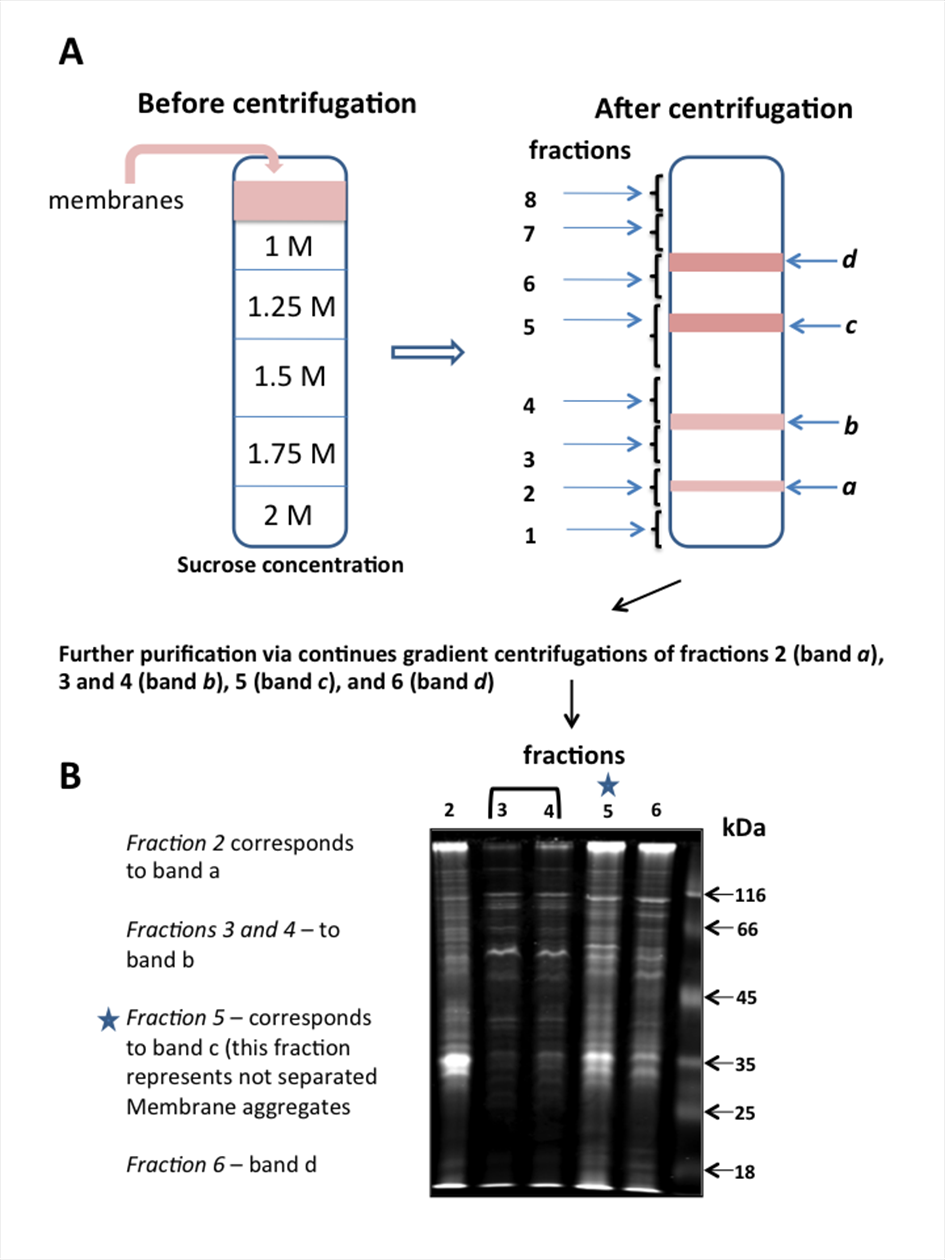

Supplement: S5 Fig — (A) Schematic diagram showing bands resulting from density gradient fractionation of membrane fractions released from cells of G. obscuriglobus lysed via sonication. On the left is the initial distribution of sucrose concentrations in the gradient before ultracentrifugation and the initial position of the total membrane mixture. On the right are the resulting bands that were visible after ultracentrifugation—fractions collected from the whole length of the gradient are indicated by numbers 1–8 and the resulting protein bands are indicated as a-d. (B) SDS-PAGE gel of continuous-gradient purified fractions corresponding to fractions 2–6 of membrane bands described in (A). Bands resulting from electrophoresis of the different membrane fractions show that purified fractions 3 and 4 (band b) contain a distinctive pattern of a limited number of proteins relative to fractions 2 (band a), 5 (band c) and 6 (band d). Fractions 1, 7, and 8 did not contain any material and were excluded from further work. Purified fractions 3 and 4 were shown to contain only ‘canoe’ membranes with pore structures via TEM of negatively stained membranes (S8 Fig). Protein fraction 5 after continuous gradient fractionation formed a “smear” band which was collected and examined under electron microscope. The collected fraction was found to contain a mixture of membranes morphologically similar to those from fractions 2, 3 (and 4), and 6, and was therefore excluded from proteome analysis. Fraction 4 after preliminary Mass-spec analysis revealed the same protein content as fraction 3, thus for the analysis of the whole protein content of the band b we used fraction 3 only. (TIF) [file pone.0169432.s009.tif]

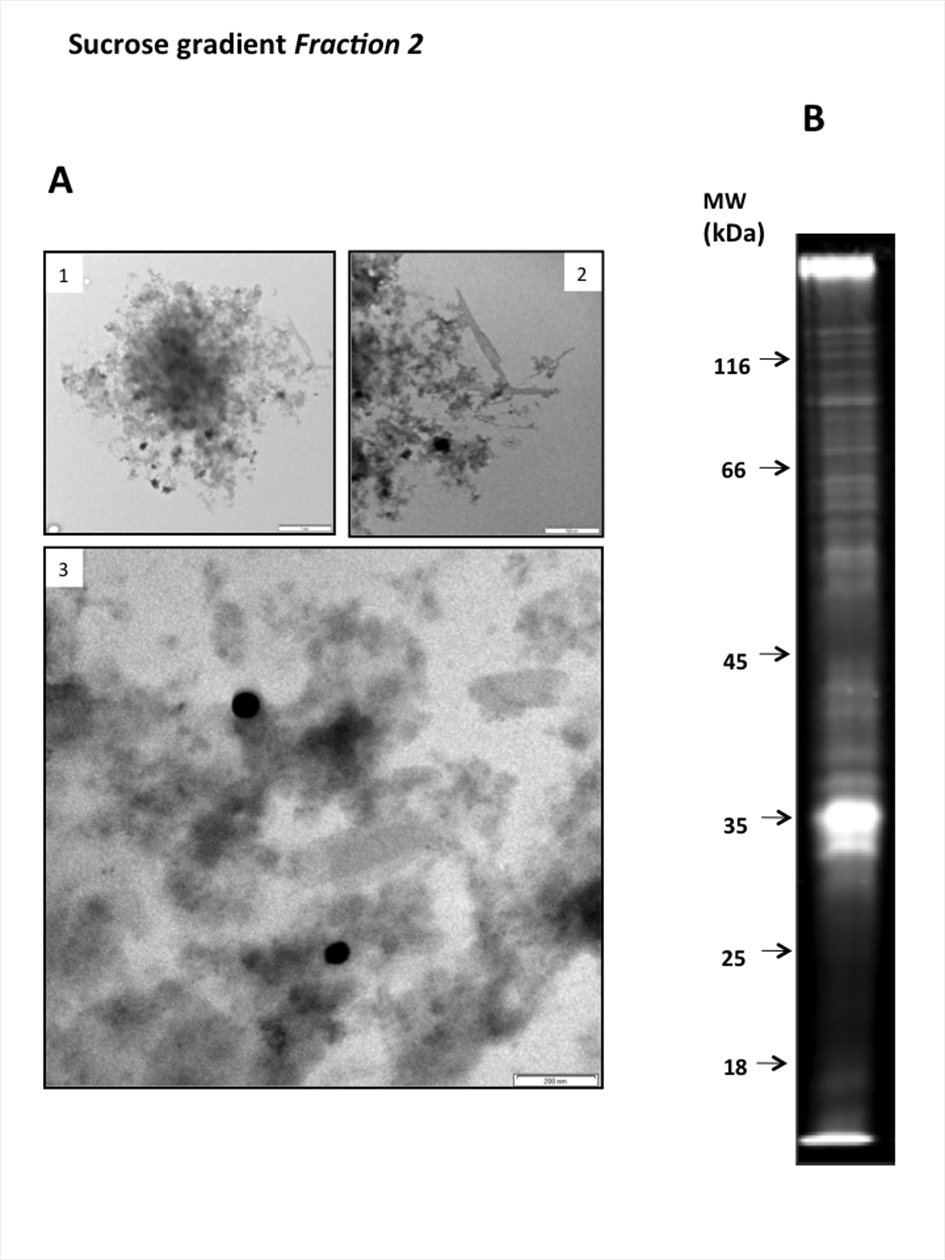

Supplement: S6 Fig — (A) (1, 2 and 3)—transmission electron micrographs of negatively stained membranes of fraction 2 (see S6 Fig) containing membranes which do not display pore complexes. Bar A1, 1 μm, Bar A2, 500 nm, Bar A3, 200 nm. (B) SDS-PAGE of membrane fraction 2 proteins. All these individual bands were cut out for proteomic analysis (for results see S1 Table). (TIF) [file pone.0169432.s010.tif]

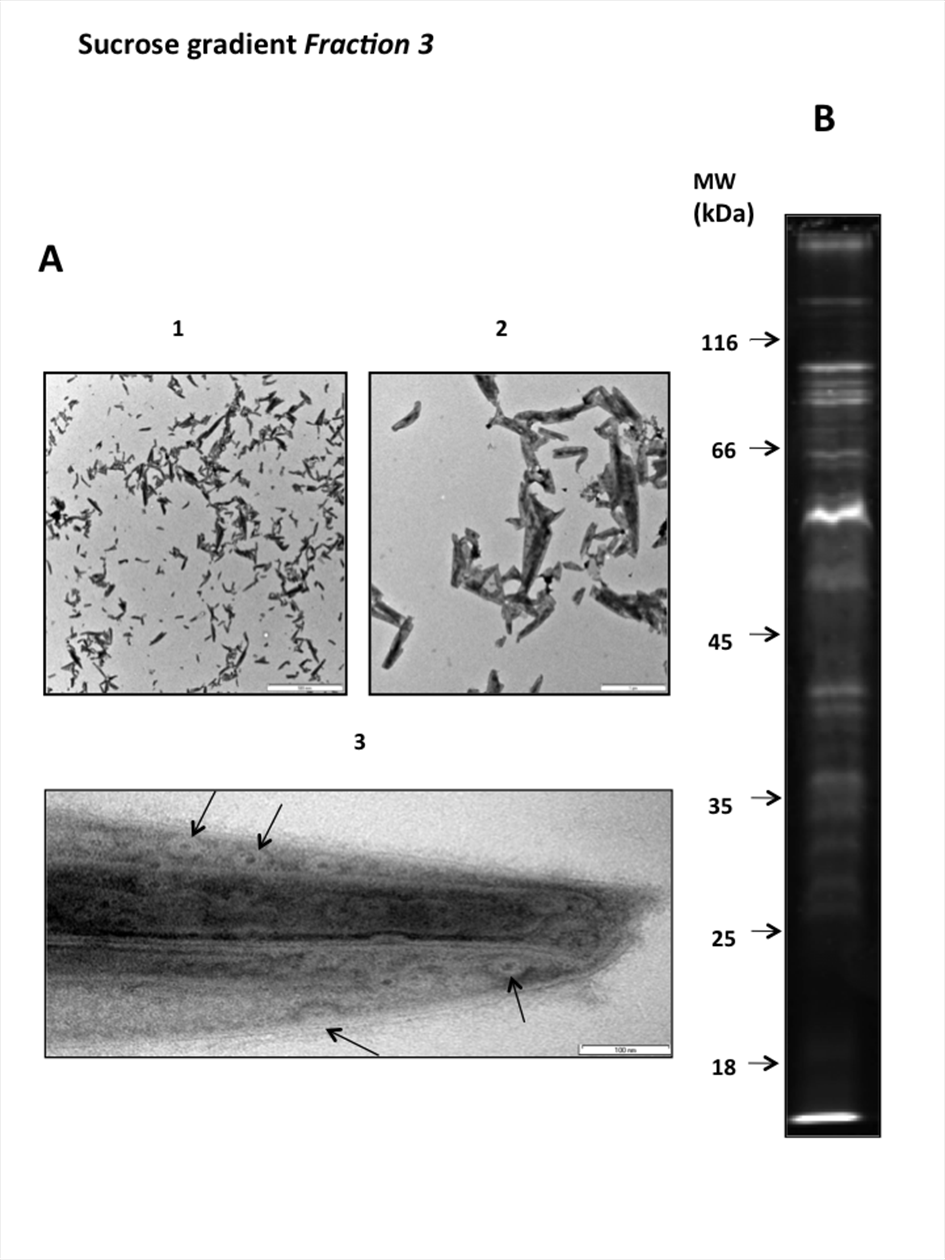

Supplement: S7 Fig — (A) TEM of negatively stained membranes of fraction 3 (see S6 Fig) containing membranes which display pore complexes. 1 and 2 show appearance of aggregates of membranes at relatively low magnification while 3 shows the characteristic ‘canoe’ shape of pore-containing membranes comprising this fraction. The enlarged view in A3 shows the typical appearance of the large pore ring structures (arrows) on these ‘canoe’-shaped membranes. Bar A1, 5 μm, Bar A2, 1 μm, Bar A3, 200 nm. (B) SDS-PAGE of membrane fraction 3 proteins. All the individual bands were cut out for proteomic analysis (for results see S1 Table). Fraction 4 contained the same ‘canoe’ shaped membranes and proteomics analysis revealed no difference between those fractions at protein level. (TIF) [file pone.0169432.s011.tif]

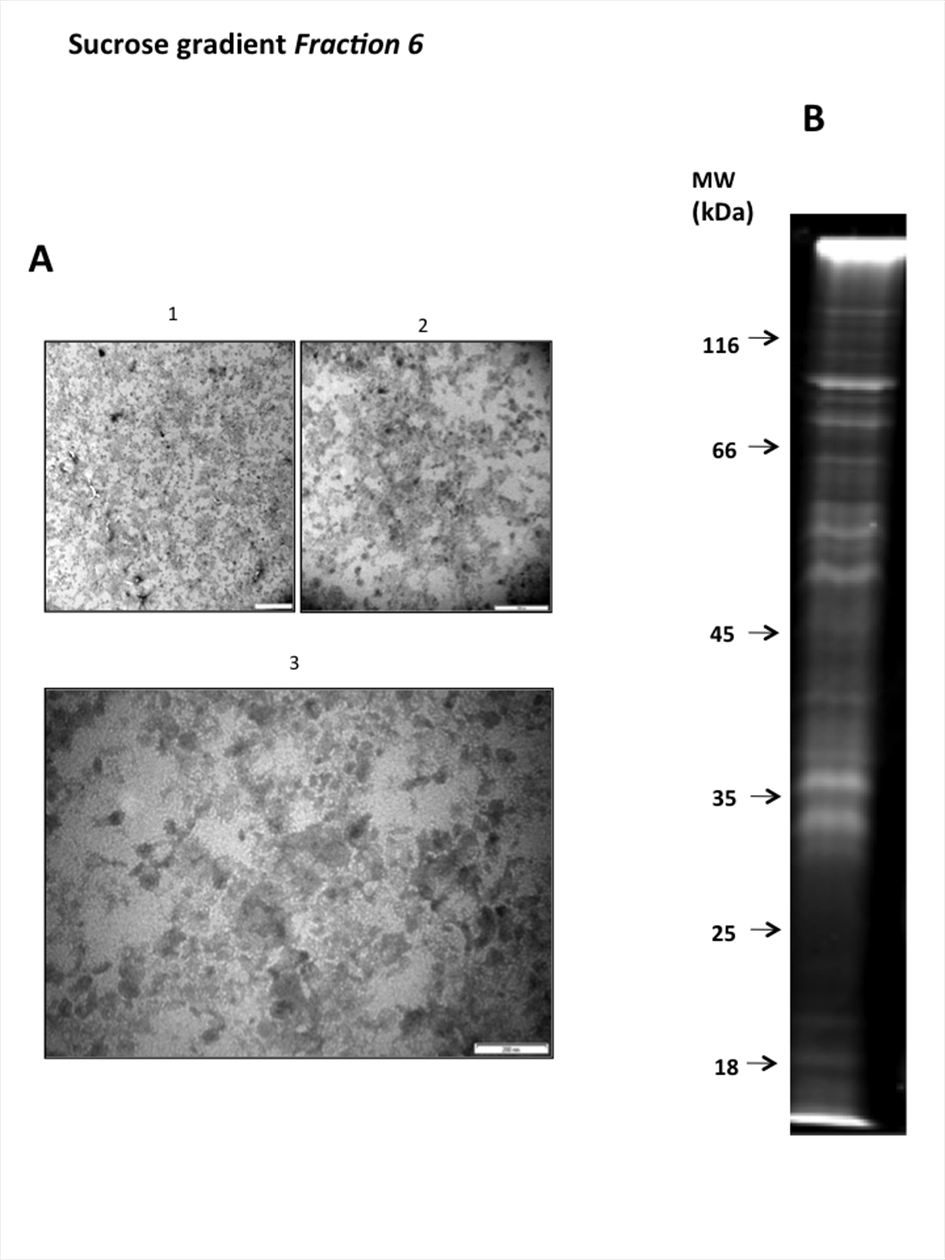

Supplement: S8 Fig — (A) Transmission electron microscopy of negatively stained membranes of fraction 6 (see S6 Fig) containing membranes which do not display pore complexes. Bar A1, 10 μm, Bar A2, 500 nm, Bar A3, 200 nm. (B) SDS-PAGE of membrane fraction 6 proteins. All the individual bands were cut out for preparation for proteomic analysis (for results see S1 Table). (TIF) [file pone.0169432.s012.tif]

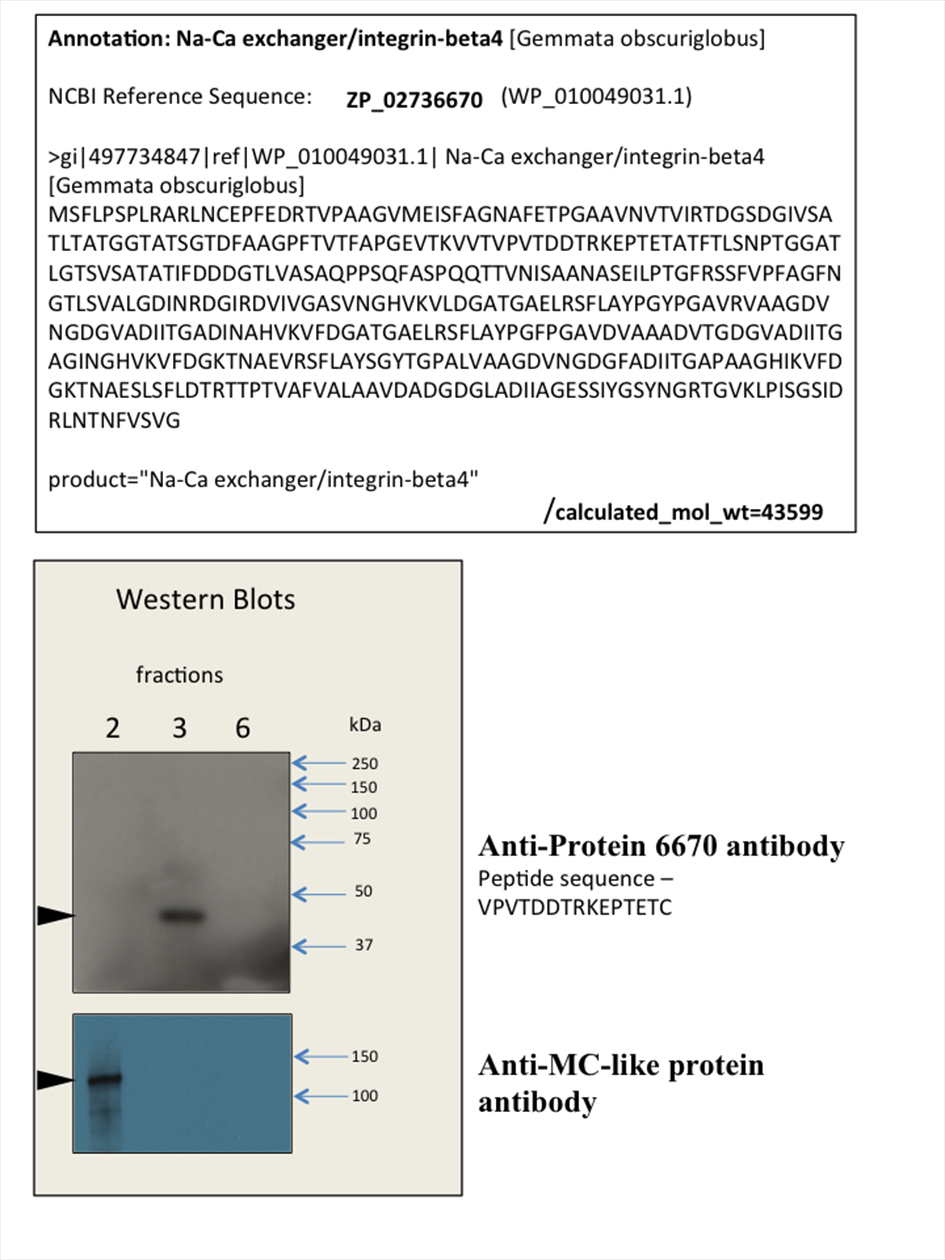

Supplement: S9 Fig — (A) Amino acid sequence of the protein annotated as Na-Ca exchanger/integrin-beta4 (NCBI Reference Sequence: ZP_02736670, later renumbered as the synonymous WP_010049031.1) was used for generation of an antibody (antibody 6670). The protein was identified by mass-spectrometry analyses as a unique protein for Fraction 3 and the full sequence was retrieved from the NCBI Database. (B) G.obscuriglobus fractions were used for testing the antibody specificity. The antibody does not react with proteins from fractions 2 and 6, and only one band was detected in fraction 3 at ca. 40–45 kDa (arrowhead), which is consistent with the predicted MW for the Na-Ca exchanger/integrin-beta4. As a control for purity of fractionations the antibody against MC-like protein was tested. The antibody recognizes specifically a protein from fraction 2 with the approximate molecular mass of 120 kDa, which is in accordance with the calculated mass for this protein. (TIF) [file pone.0169432.s013.tif]

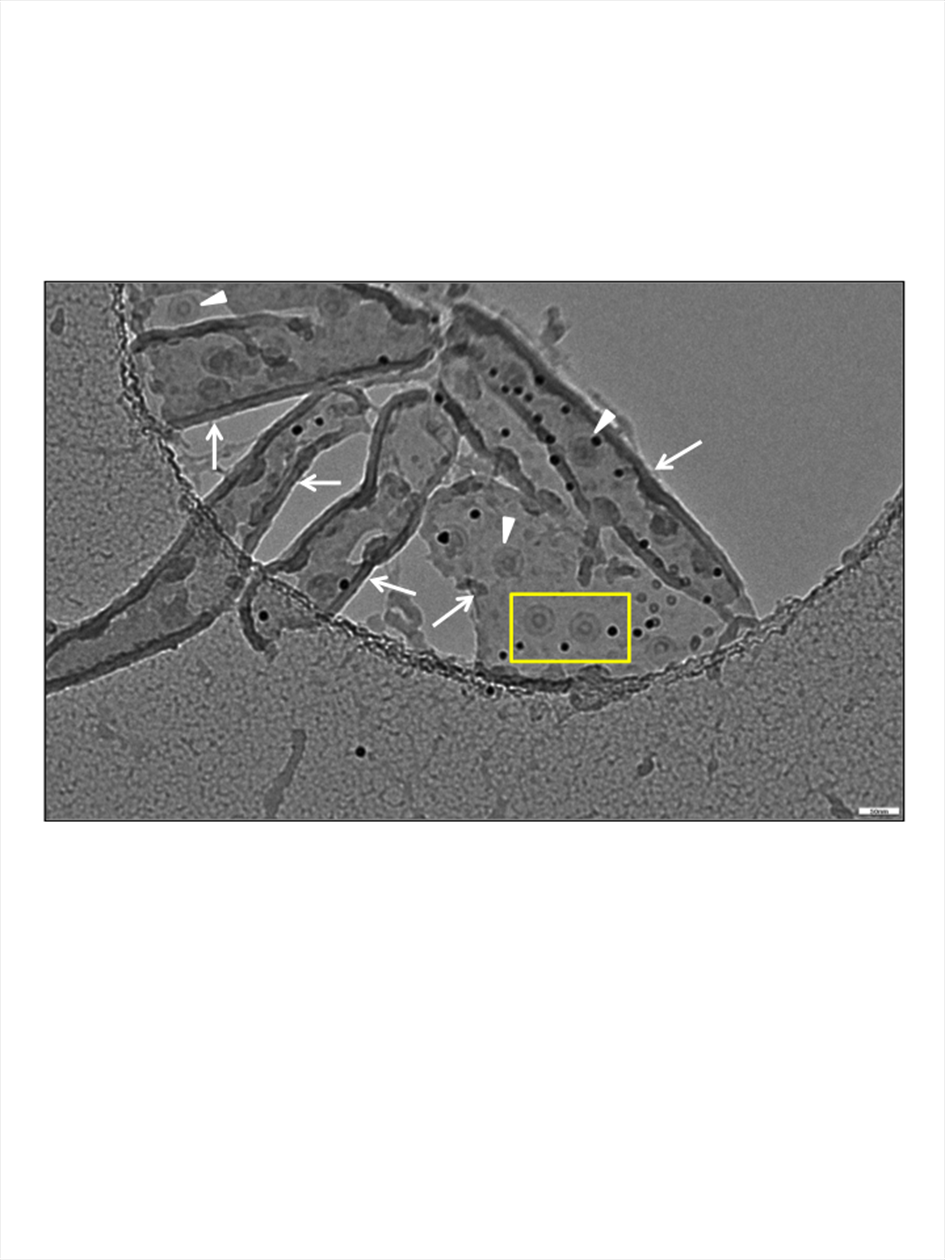

Supplement: S10 Fig — Cryo-EM image of frozen-hydrated sucrose-purified membranes from fraction 3 isolated from lysed cell preparation by density gradient centrifugation. Membrane sheets are indicated by arrows and large pore structures are marked by arrowheads. The two pores in the boxed region are seen in Fig 4C. (TIF) [file pone.0169432.s014.tif]

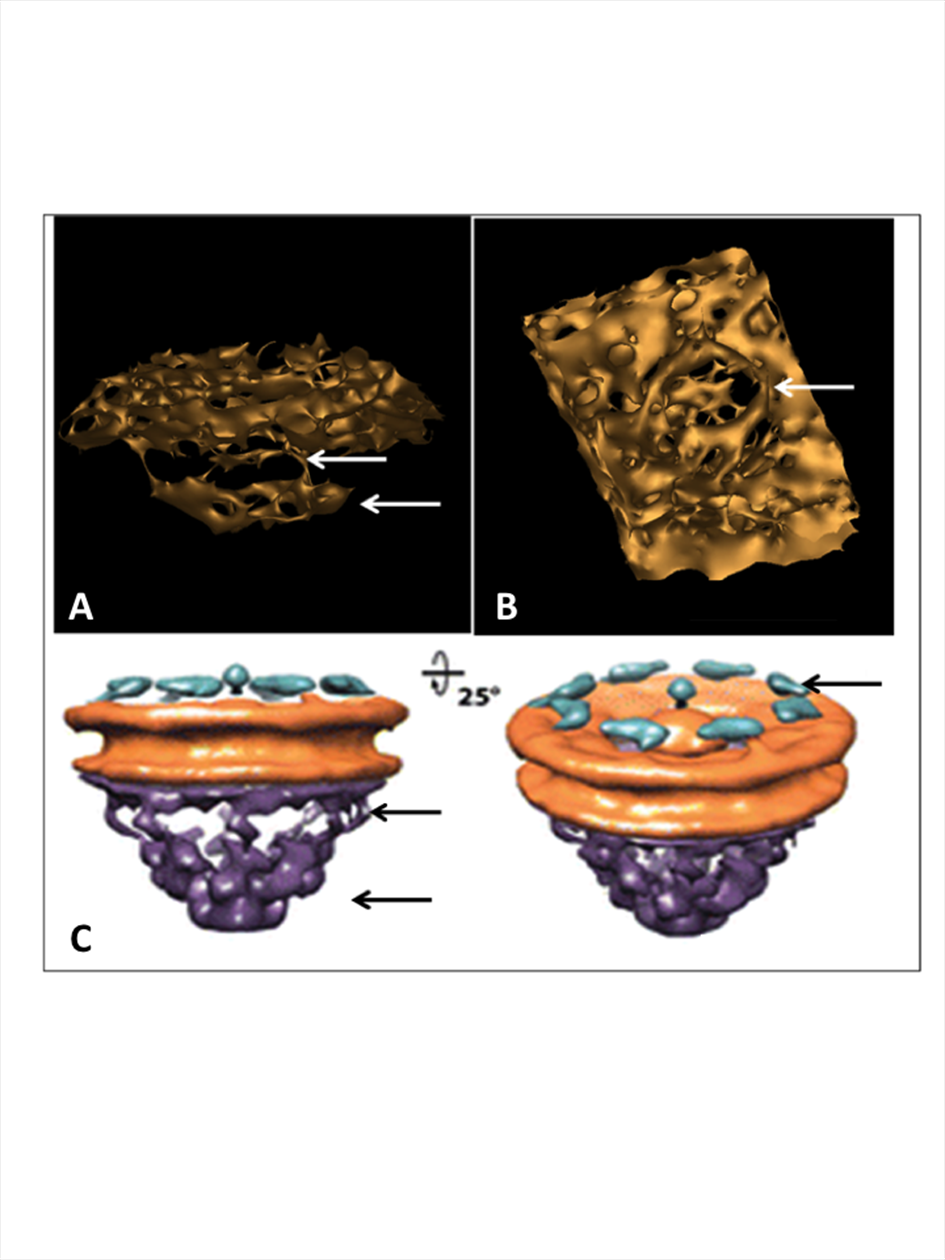

Supplement: S11 Fig — (A) and (B) Reconstruction of architecture of a single pore from two different angles. In panel (A), a side view of the pore displays the basket structure with a series of struts (arrows) connecting with the main pore rings. In panel (B), a top view shows the ring-like element (arrow) of the main part of the pore and a central plug structure is visible within the pore connected to the ring’s inner rim via spokes. The same major pore structural elements (plug, ring and spokes) are indicated in the eukaryote nuclear pore shown in S11C Fig and the pores are shown at similar angles (C) The image in (C) represents a cryo-electron tomographic reconstruction of the Dictyostelium discoideum nuclear pore complex published previously by Beck et al. [26]. (TIF) [file pone.0169432.s015.tif]

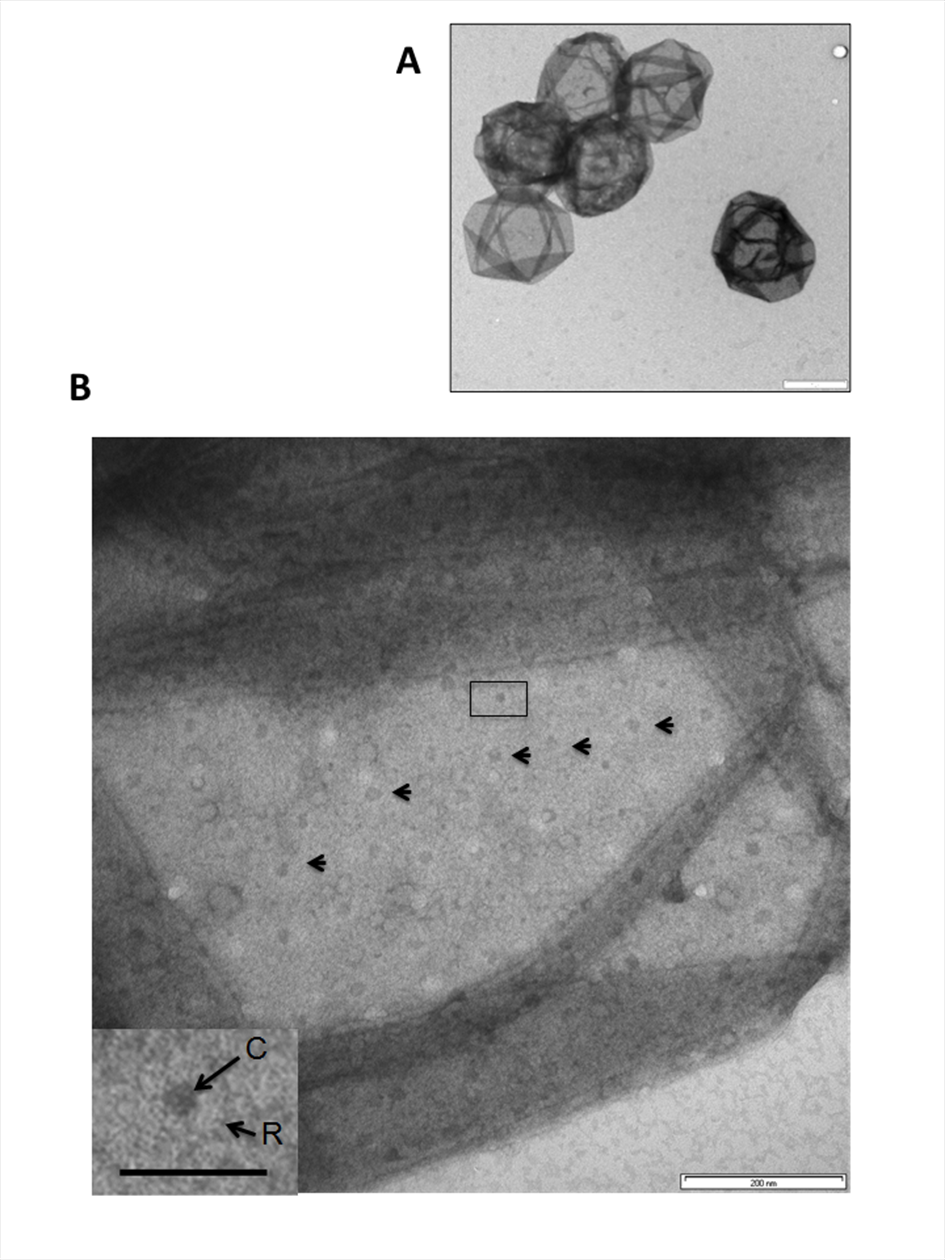

Supplement: S12 Fig — (A) TEM of cell walls of G.obscuriglobus isolated via boiling of bacteria in 10% SDS for 1hr. Bar, 2 μm. (B) One of the cell walls of G.obscuriglobus with clearly recognizable crateriform structures (arrowheads). The electron dense core regions are variable in shape. Bar, 200 nm. Inset: an enlarged view of the boxed area in A showing a single crateriform structure with central electron-dense core surrounded by a single electron-transparent ring. There is no indication of division of the ring region into an inner and outer ring. Bar, 50 nm. (TIF) [file pone.0169432.s016.tif]

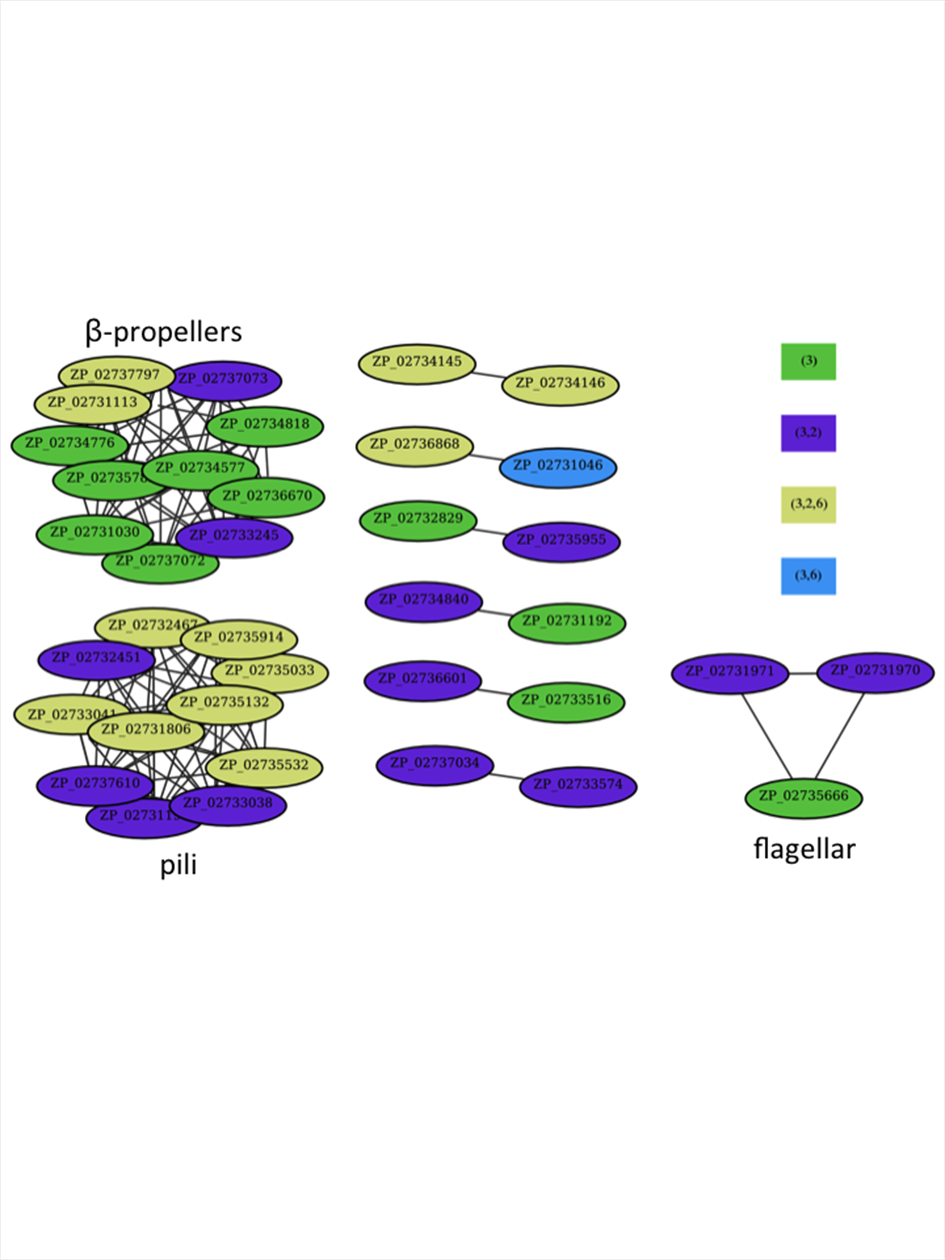

Supplement: S13 Fig — All 128 proteins associated with the membrane fractions were clustered using VisBLAST (E = 0.001, i = 2). Proteins are identified by Genbank accession numbers. Lines indicate detectable sequence similarity between proteins. Colour key indicates membrane fractions in which proteins were detected. The 91 singleton proteins that did not show any significant sequence similarity to the other proteins are listed in S3 Table. The cluster in the top left corner (cluster 1) corresponds to the cluster containing fraction 3 proteins from S14 Fig. Structural modelling using Phyre2 indicates that constituents of this cluster carry beta-propeller folds (Fig 7C and S4 Table). The cluster on the bottom left is dominated by pili proteins (cluster 2) (see also S18 Fig and S5 Table). (TIF) [file pone.0169432.s017.tif]

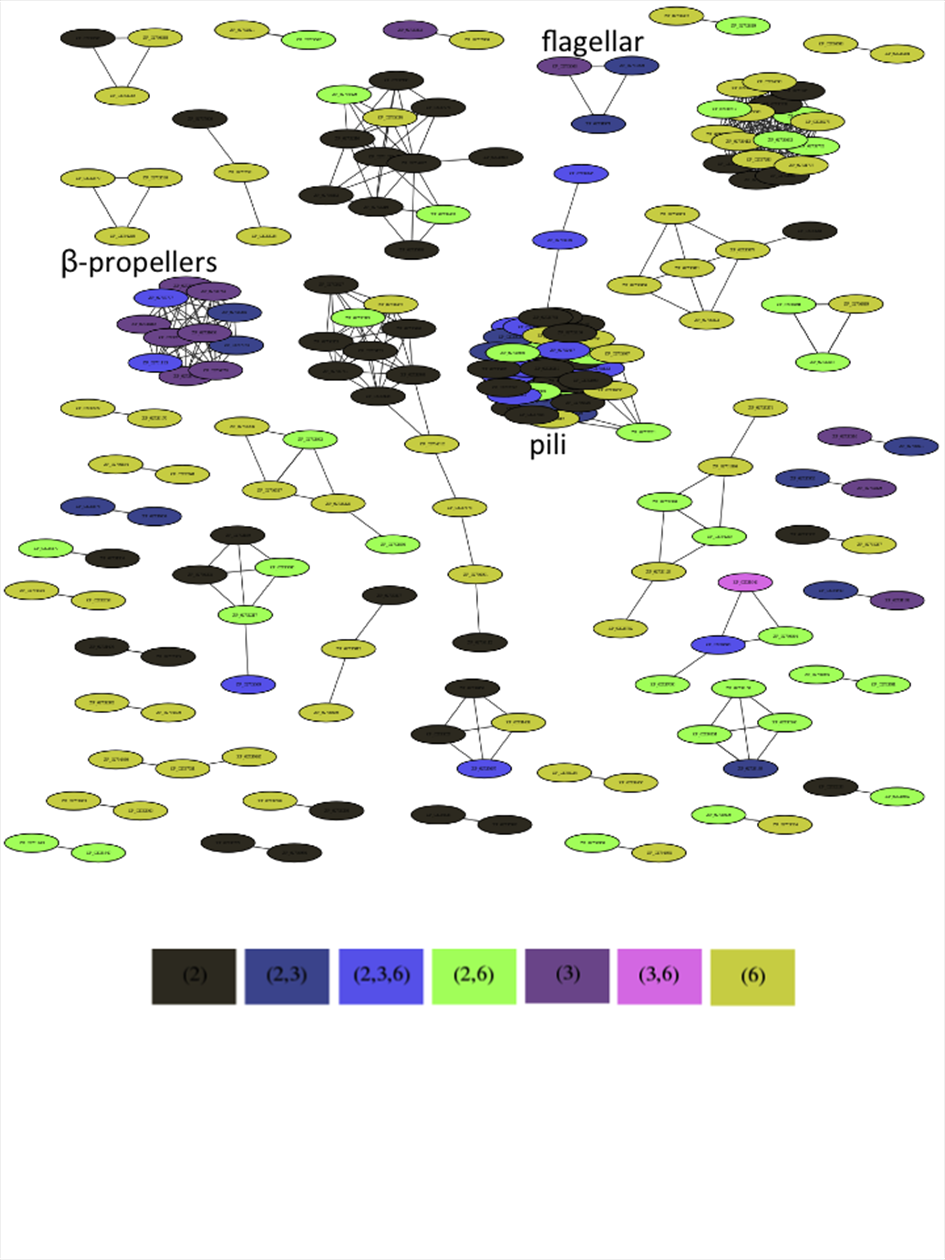

Supplement: S14 Fig — All 512 identified in our proteomics analysis were clustered using VisBLAST (E = 0.001, i = 2). This revealed several large clusters, though only one of these contained proteins specific to fraction 3 (cluster 1). Proteins are identified by Genbank accession numbers. Lines indicate detectable sequence similarity between two proteins. Colour key indicates in which membrane fractions proteins were detected. (TIF) [file pone.0169432.s018.tif]

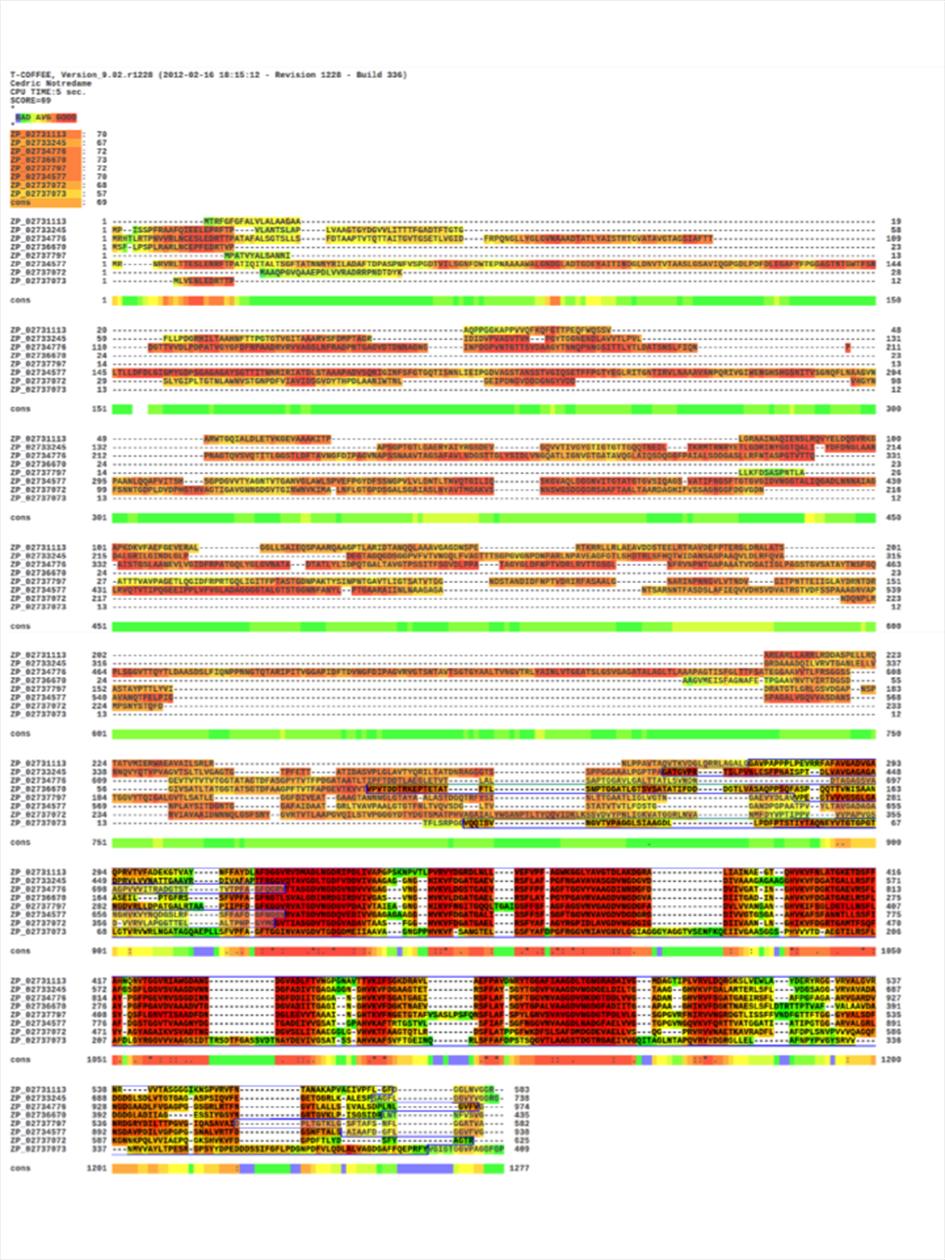

Supplement: S15 Fig — The multiple alignment of the 8 proteins from cluster 1 (top left cluster in S13 Fig and S4 Table) that gave significant structure predictions using Phyre2 is shown. The alignment was made using MAFFT, option L-ins-i, and was evaluated using the T-Coffee CORE server (see Supplementary Text for details). The modelled structures are indicated by the highlighted part of the alignment at the C-terminal end. Structural models (Fig 3) correspond to the conserved part of the sequences. (TIF) [file pone.0169432.s019.tif]

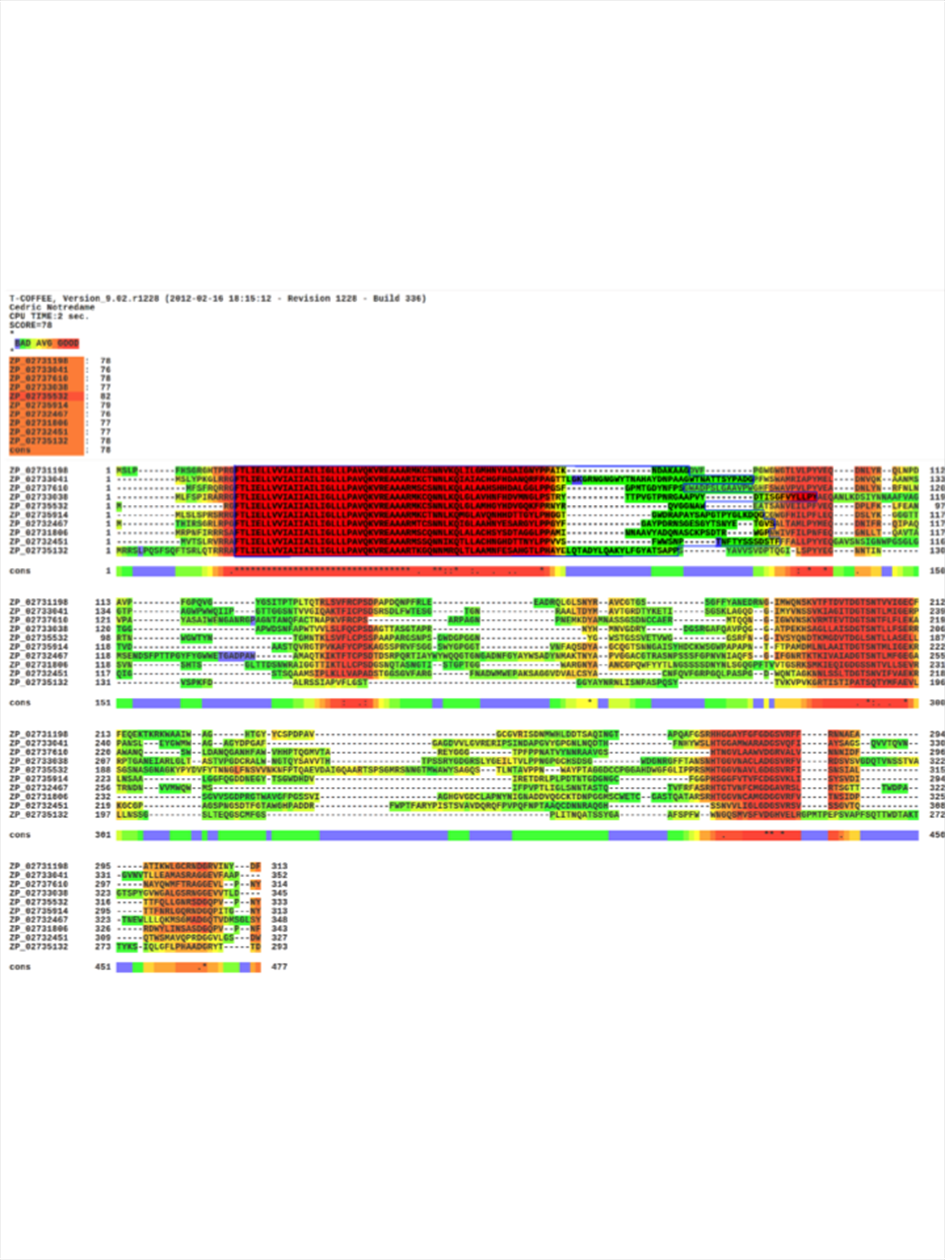

Supplement: S16 Fig — The 10 pili proteins in cluster 2 (bottom left in S13 Fig and S5 Table) that gave significant structure predictions using Phyre2 were aligned using MAFFT, option L-ins-i, and the alignment was evaluated using the T-Coffee CORE server (see text for details). The predicted structures are all clearly located in the conserved subsequence in the N-terminal end of the alignment (see the highlighted part). (TIF) [file pone.0169432.s020.tif]

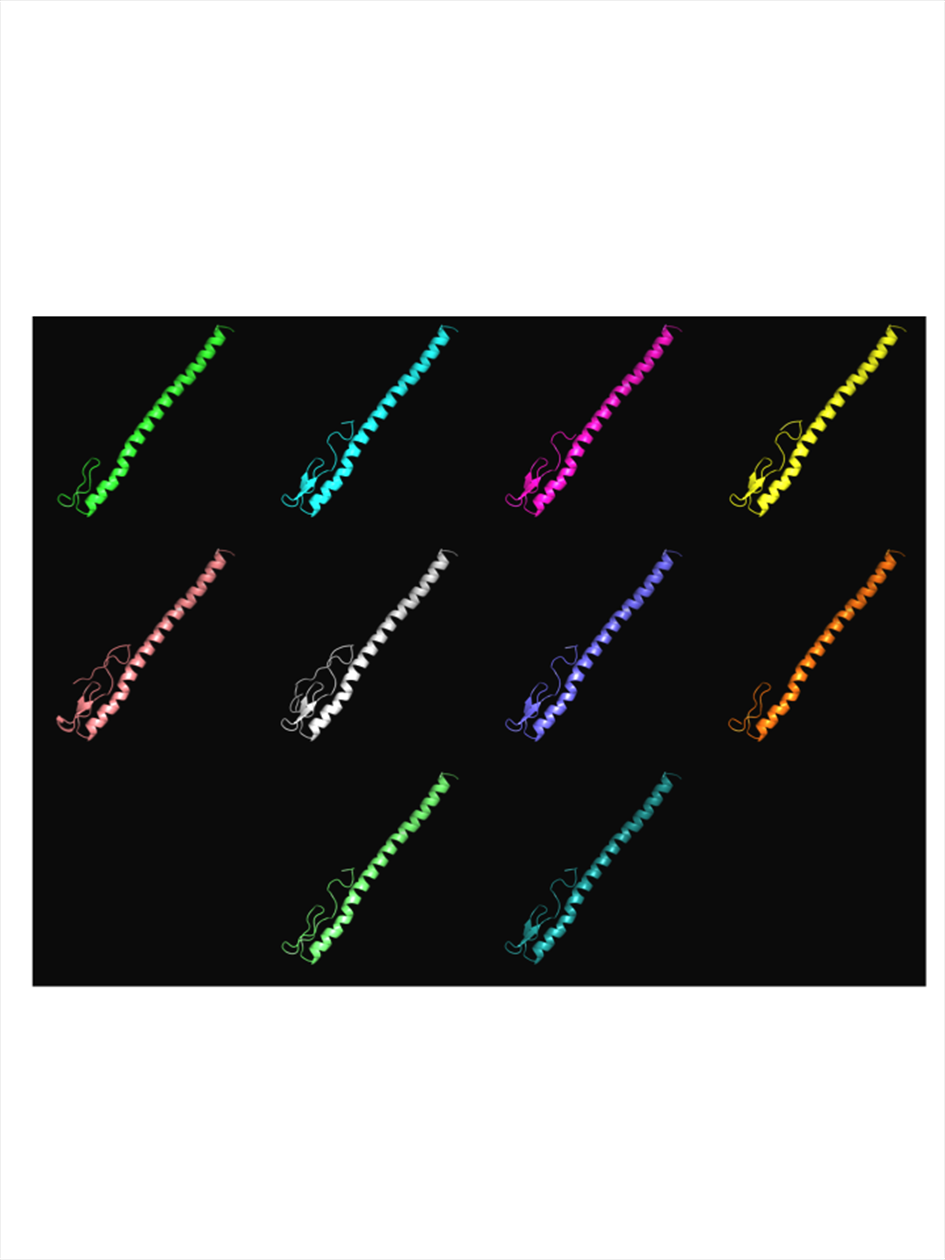

Supplement: S17 Fig — Using PyMOL, the predicted structures for the 11 proteins from membrane fraction 3 that were clustered together and shared a pili-like structure were visualized. None of these are unique to fraction 3 (S5 Table). The structures are for (from left to right and top to bottom): ZP_02731198, ZP_02731806, ZP_02732451, ZP_02732467, ZP_02733038, ZP_02733041, ZP_02735033, ZP_02735132, ZP_02735532, ZP_02735914 and ZP_02737610. (TIF) [file pone.0169432.s021.tif]

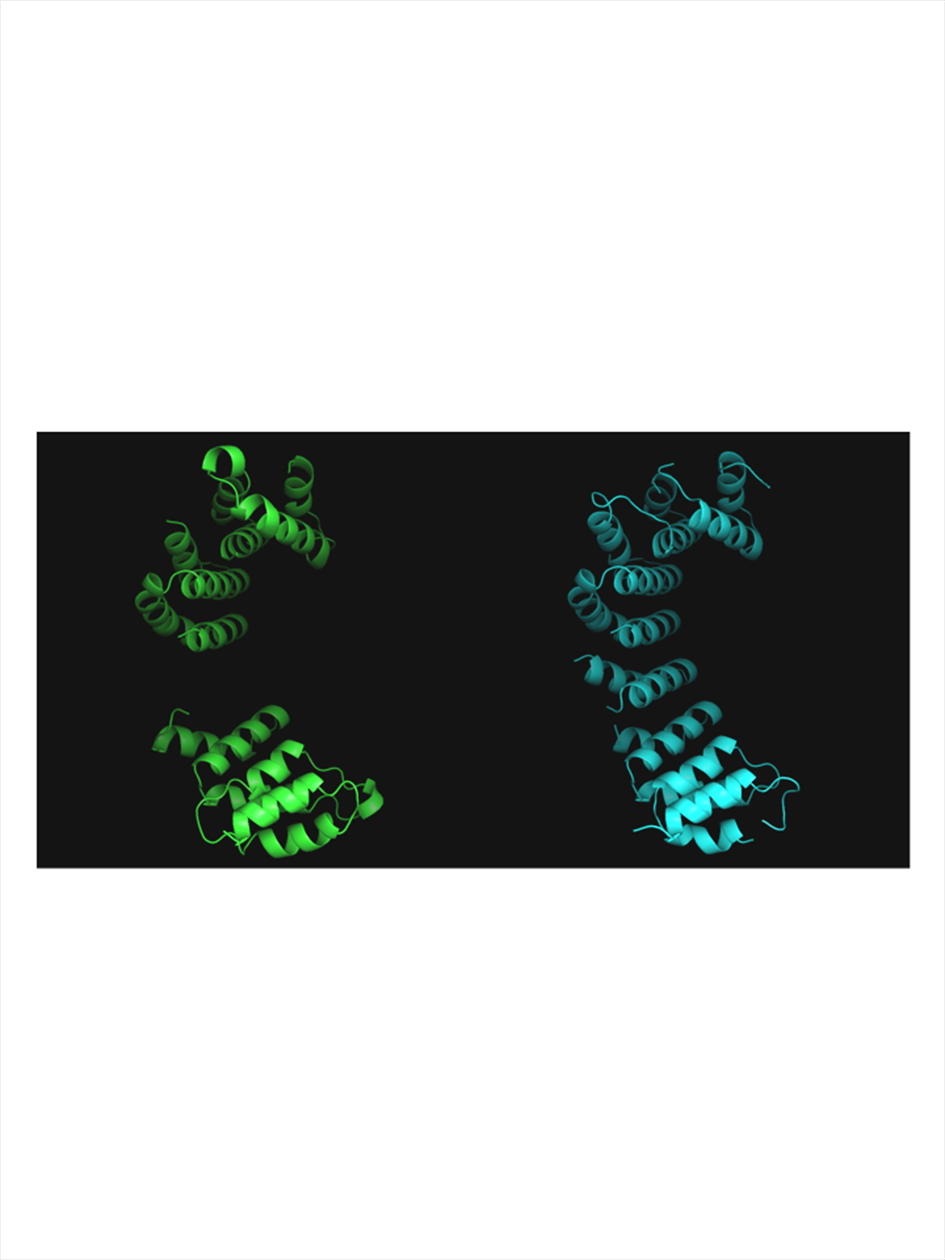

Supplement: S18 Fig — Two proteins showed a potential α-solenoid structure with stacked α-helices. Left: ZP_02735673 (constituent of fraction 2 and fraction 3) and right: ZP_02736511, unique to pore-containing membrane fraction 3. (TIF) [file pone.0169432.s022.tif]

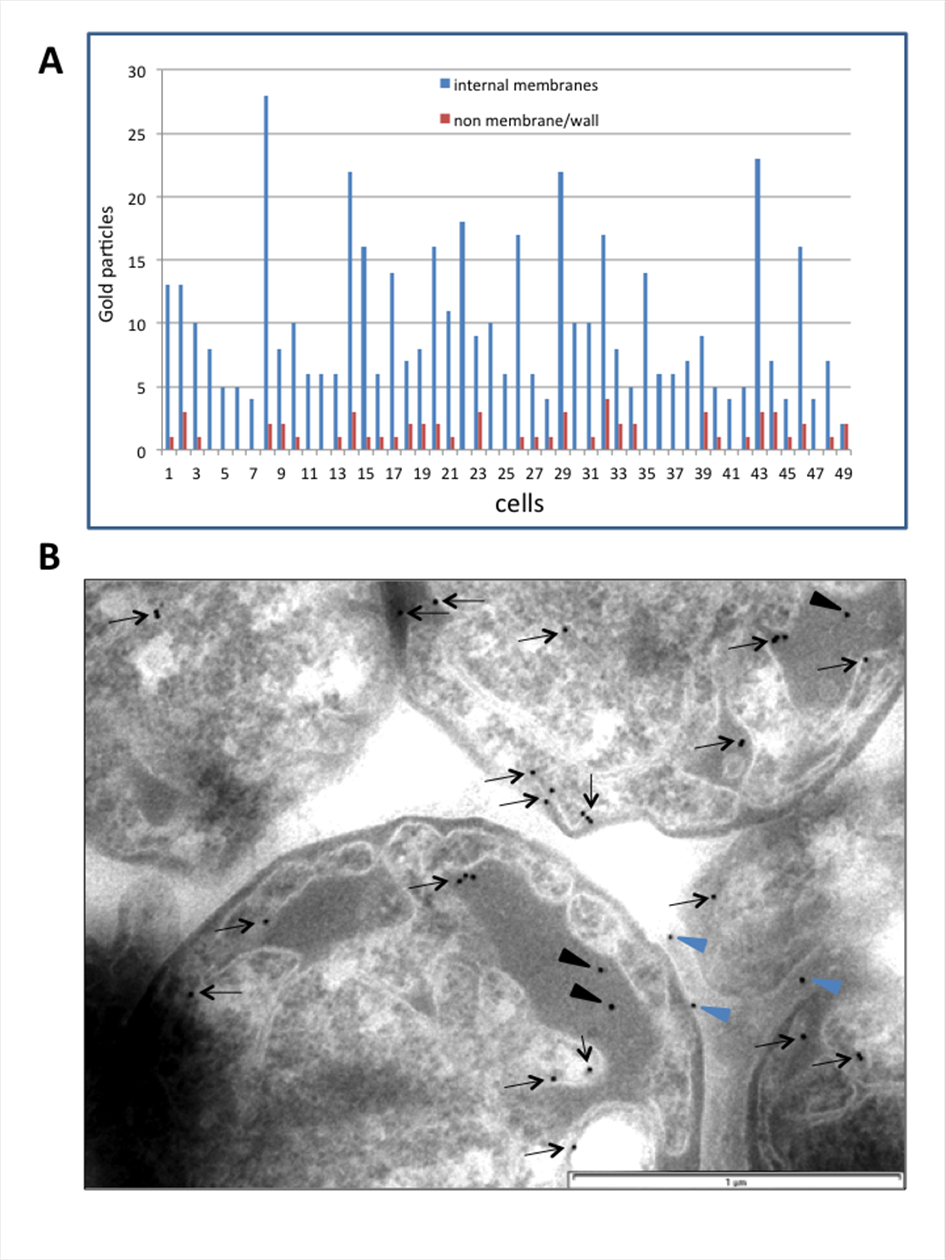

Supplement: S19 Fig — (A)Distribution of gold particles within Gemmata obscuriglobus cells. The bars represent a number of particles associated with the intracytoplasmic membranes (blue bars) vs with no visible association with the membranes (red bars). A total of 50 cells were used for the counting; 549 particles were found as associated with the membranes and 60 as not associated. The particles were counted according to the description in (B). (B) An example of gold particle distribution within the cells of Gemmata obscuriglobus, labelled with 6670 antibody and then with 10 nm gold protein A. The majority of the particles were found associated with the intracytoplasmic membrane (black arrows). The particles were considered as membrane-associated if the distance between the membrane and the particle did not exceed 20 nm. Blue arrowheads indicate particles which were counted as a background or cell wall-associated, black arrowheads show non-membrane associated particles. Bar, 1 μm. (TIF) [file pone.0169432.s023.tif]
