# Supplementary material for: Nuclear Pore-Like Structures in a Compartmentalized Bacterium
Source: PLoS One. 2017 Feb 1;12(2):e0169432. doi: 10.1371/journal.pone.0169432 (PMC5287468; doi:10.1371/journal.pone.0169432)
Supplement: S1 Table — (DOC) [file pone.0169432.s024.doc]

**S1 Table. Proteins identified in membrane fractions by MALDI-TOF.**

| **Fraction 2** (if protein found in other fractions it is indicated in brackets) | **Protein name** | **NCBI accession numbers** | **Calculated molecular weight (Da)*** | **MOWSE score** | **Peptides matched** | **Sequence coverage (%)** |
| --- | --- | --- | --- | --- | --- | --- |
| 2 | hypothetical protein GobsU_11075 (MC-like protein) | ZP_02732338 | 124479 | 969 | 18 | 19.3 |
| 2 (6) | 4Fe-4S ferredoxin, iron-sulfur binding domain protein | ZP_02733602 | 122387 | 1335 | 18 | 26.2 |
| 2 (3,6) | hypothetical protein GobsU_29906 | ZP_02736062 | 76943 | 1341 | 71 | 40.8 |
| 2 (3) | type IV fimbrial assembly protein PilB | ZP_02734149 | 70569 | 1068 | 39 | 30.3 |
| 2 (6) | probable DNA-directed RNA polymerase alpha chain | ZP_02736955 | 51701 | 762 | 15 | 39.1 |
| 2 (6) | F0F1 ATP synthase subunit beta | ZP_02731447 | 52132 | 939 | 14 | 40.3 |
| 2 (3,6) | hypothetical protein GobsU_20253 | ZP_02734146 | 26677 | 693 | 74 | 53.9 |
| 2 (6) | oxidoreductase, short chain dehydrogenase/reductase family protein | ZP_02733345 | 24580 | 509 | 23 | 42.8 |
| 2 | short chain dehydrogenase | ZP_02732964 | 28767 | 258 | 13 | 18.4 |
| 2 (3,6) | hypothetical protein GobsU_27236 | ZP_02735532 | 35749 | 883 | 94 | 50.8 |
| 2 (6) | hypothetical protein GobsU_17456 | ZP_02733593 | 12531 | 232 | 3 | 31.6 |
| 2 (6) | hypothetical protein GobsU_11415 | ZP_02732406 | 14995 | 394 | 5 | 36.6 |
| 2 (6) | DNA-directed RNA polymerase beta chain | ZP_02733087 | 141275 | 760 | 13 | 15.4 |
| 2 (6) | DNA-directed RNA polymerase beta chain | ZP_02733088 | 164212 | 514 | 14 | 9.2 |
| 2 (6) | hypothetical protein GobsU_34912 | ZP_02737058 | 127649 | 1141 | 18 | 17.9 |
| 2 | putative exported protease | ZP_02737000 | 118801 | 486 | 10 | 13.9 |
| 2 | signal transduction histidine kinase with CheB and CheR activity | ZP_02734469 | 104719 | 480 | 8 | 12.9 |
| 2 (6) | hypothetical protein GobsU_35960 | ZP_02737261 | 103499 | 446 | 9 | 11.4 |
| 2 | dihydrolipoamide dehydrogenase | ZP_02733335 | 50314 | 706 | 10 | 31.4 |
| 2 | hypothetical protein GobsU_14594 | ZP_02733027 | 56438 | 326 | 5 | 12.5 |
| 2 | hypothetical protein GobsU_25171 | ZP_02735122 | 52187 | 280 | 5 | 14.3 |
| 2 | hypothetical protein GobsU_14434 | ZP_02732995 | 52892 | 274 | 8 | 22 |
| 2 (6) | Cobalamin synthesis protein/P47K | ZP_02730707 | 41940 | 201 | 7 | 13 |
| 2 | glycogen synthase | ZP_02733261 | 56208 | 220 | 3 | 11.2 |
| 2 (6) | multidrug efflux system, HlyD family subunit | ZP_02734259 | 47627 | 199 | 4 | 11.2 |
| 2 (6) | 6-phosphogluconate dehydrogenase | ZP_02734911 | 52647 | 155 | 3 | 11.2 |
| 2 | hypothetical protein GobsU_04689 | ZP_02731070 | 52224 | 165 | 7 | 9.8 |
| 2 | hypothetical protein GobsU_20468 | ZP_02734189 | 25555 | 206 | 2 | 15.4 |
| 2 (3) | Uridylate kinase | ZP_02736756 | 26457 | 425 | 20 | 34.1 |
| 2 (6) | Sulphate transport system permease protein 1 | ZP_02732762 | 39652 | 326 | 16 | 19.4 |
| 2 (3,6) | hypothetical protein GobsU_06435 | ZP_02731416 | 103608 | 654 | 15 | 17 |
| 2 (3,6) | aconitate hydratase 1 | ZP_02730459 | 98013 | 514 | 10 | 15.5 |
| 2 (6) | protein-export membrane protein secD | ZP_02733003 | 123971 | 699 | 10 | 11.8 |
| 2 (3,6) | putative peptidase | ZP_02736310 | 42703 | 766 | 62 | 39 |
| 2 (3,6) | hypothetical protein GobsU_20233 | ZP_02734142 | 48877 | 686 | 56 | 34 |
| 2 | methyltransferase | ZP_02734785 | 43932 | 352 | 18 | 11 |
| 2 | chorismate mutase | ZP_02736115 | 41559 | 346 | 13 | 11 |
| 2 (6) | methylcitrate synthase | ZP_02732426 | 42530 | 250 | 14 | 11 |
| 2 (3) | flagellar basal body rod protein FlgG | ZP_02731970 | 27437 | 418 | 12 | 29.7 |
| 2 (6) | putative ABC transporter ATP-binding protein | ZP_02736693 | 34576 | 1208 | 93 | 67.9 |
| 2 (6) | hypothetical protein GobsU_14709 | ZP_02733050 | 37118 | 312 | 25 | 20.9 |
| 2 (3,6) | 50S ribosomal protein L1 | ZP_02733084 | 30488 | 343 | 35 | 21.5 |
| 2 | succinic semialdehyde dehydrogenase | ZP_02737897 | 17115 | 159 | 8 | 30.2 |
| 2 (6) | cobalt-zinc-cadmium resistance protein | ZP_02733511 | 54820 | 194 | 5 | 11.7 |
| 2 | bifunctional GMP synthase/glutamine amidotransferase protein | ZP_02737792 | 56752 | 216 | 9 | 11.3 |
| 2 | branched-chain amino acid transport ATP-binding protein | ZP_02731798 | 25852 | 267 | 11 | 23.4 |
| 2 | Streptomyces cyclase/dehydrase | ZP_02734441 | 20855 | 174 | 10 | 23 |
| 2 (6) | ketol-acid reductoisomerase | ZP_02737871 | 38760 | 457 | 14 | 28.7 |
| 2 (3,6) | hypothetical protein GobsU_20643 | ZP_02734224 | 92712 | 526 | 15 | 12.7 |
| 2 (6) | hypothetical protein GobsU_04644 | ZP_02731061 | 47023 | 306 | 17 | 8 |
| 2 (3,6) | hypothetical protein GobsU_31654 | ZP_02736410 | 87810 | 452 | 10 | 11.5 |
| 2 (3,6) | oxidoreductase domain protein | ZP_02735697 | 47995 | 1426 | 157 | 113 |
| 2 (6) | serine proteinase, HtrA/DegQ/DegS family | ZP_02735252 | 42708 | 236 | 11 | 11 |
| 2 | xylose isomerise domain protein TIM barrel | ZP_02734509 | 30645 | 110 | 4 | 11.5 |
| 2 (3,6) | translation-associated GTPase | ZP_02730857 | 39663 | 200 | 7 | 6 |
| 2 (6) | elongation factor Tu | ZP_02733080 | 47629 | 359 | 9 | 16.4 |
| 2 (6) | pyruvate dehydrogenase complex, dihydrolipoamide acetyltransferase E2 component | ZP_02735609 | 56948 | 483 | 18 | 17.4 |
| 2 (6) | ATP synthase F1, alpha subunit | ZP_02731443 | 62190 | 688 | 24 | 23.5 |
| 2 (6) | hypothetical protein GobsU_23532 | ZP_02734797 | 61448 | 711 | 25 | 22.2 |
| 2 (3,6) | Flagellar FliF M-ring protein | ZP_02730987 | 54974 | 136 | 11 | 7.5 |
| 2 (6) | Ferredoxin | ZP_02732656 | 63129 | 523 | 30 | 20.2 |
| 2 (3,6) | hypothetical protein GobsU_04904 | ZP_02731113 | 61669 | 881 | 47 | 29.3 |
| 2 (6) | sigma-54 dependent transcriptional regulator/response regulator | ZP_02732401 | 53500 | 754 | 31 | 30.5 |
| 2 (3) | hypothetical protein GobsU_28805 | ZP_02735845 | 67828 | 775 | 35 | 25.4 |
| 2 (6) | RNA binding S1 domain protein | ZP_02737093 | 118263 | 588 | 24 | 12.7 |
| 2 (6) | sialic acid-specific 9-O-acetylesterase | ZP_02737096 | 57483 | 960 | 49 | 32 |
| 2 (6) | hypothetical protein GobsU_34522 | ZP_02736980 | 59238 | 472 | 12 | 18.4 |
| 2 (6) | hypothetical protein GobsU_14549 | ZP_02733018 | 54836 | 467 | 18 | 18.1 |
| 2 (3,6) | hypothetical protein GobsU_38147 | ZP_02737694 | 46334 | 350 | 36 | 17.9 |
| 2 (6) | WD-40 repeat | ZP_02737050 | 72113 | 472 | 15 | 12 |
| 2 (6) | hypothetical protein GobsU_16609 | ZP_02733426 | 56115 | 201 | 11 | 8.1 |
| 2 (6) | Signal Transduction Histidine Kinases (STHK) | ZP_02734429 | 58312 | 663 | 12 | 28.2 |
| 2 (6) | fumarate hydratase | ZP_02735455 | 54808 | 194 | 7 | 13.5 |
| 2 (6) | succinate dehydrogenase flavoprotein subunit | ZP_02737002 | 70751 | 370 | 20 | 14 |
| 2 (6) | heat shock protein GroEL | ZP_02737557 | 58429 | 167 | 6 | 9.9 |
| 2 (6) | probable chemotaxis transducer | ZP_02735177 | 59822 | 935 | 36 | 35.6 |
| 2 (6) | hypothetical protein GobsU_24941 | ZP_02735076 | 56127 | 258 | 7 | 15.5 |
| 2 (6) | hypothetical protein GobsU_27341 | ZP_02735553 | 61963 | 172 | 4 | 9.4 |
| 2 (3,6) | hypothetical protein GobsU_38668 | ZP_02737797 | 58509 | 755 | 60 | 26.5 |
| 2 (6) | 30S ribosomal protein S1 | ZP_02730364 | 57995 | 729 | 27 | 28.6 |
| 2 (3,6) | 30S ribosomal protein S1 | ZP_02736868 | 69112 | 198 | 3 | 8.4 |
| 2 (6) | heat shock protein 70 family protein | ZP_02735562 | 66391 | 593 | 22 | 23.2 |
| 2 (6) | 2-isopropylmalate synthase | ZP_02732952 | 57498 | 324 | 17 | 14.8 |
| 2 (6) | PrkA AAA domain protein | ZP_02733099 | 79001 | 1430 | 82 | 33.5 |
| 2 (6) | hypothetical protein GobsU_19384 | ZP_02733973 | 47872 | 555 | 19 | 24.6 |
| 2 (6) | hypothetical protein GobsU_20458 | ZP_02734187 | 63184 | 934 | 39 | 35 |
| 2 (6) | flagellin FliC | ZP_02737314 | 61923 | 1470 | 90 | 45 |
| 2 (6) | type IV fimbrial assembly protein PilB | ZP_02734151 | 63301 | 663 | 33 | 29.2 |
| 2 (6) | trigger factor | ZP_02733942 | 55785 | 1081 | 57 | 27 |
| 2 (6) | hypothetical protein GobsU_21120 | ZP_02734319 | 66262 | 130 | 3 | 5.3 |
| 2 (6) | alkaline phosphatase | ZP_02732407 | 66257 | 277 | 8 | 11.2 |
| 2 (6) | 60 kDa chaperonin | ZP_02736491 | 61375 | 679 | 33 | 26.6 |
| 2 (6) | hypothetical protein GobsU_30770 | ZP_02736234 | 68086 | 306 | 10 | 10 |
| 2 (6) | glycyl-tRNA synthetase | ZP_02736496 | 63211 | 198 | 7 | 11.2 |
| 2 | hypothetical protein GobsU_15760 | ZP_02733259 | 66647 | 426 | 15 | 15.4 |
| 2 | hypothetical protein GobsU_34902 | ZP_02737056 | 63883 | 582 | 16 | 18.3 |
| 2 (3,6) | glutamine synthetase, catalytic region | ZP_02737578 | 79979 | 1309 | 78 | 37.1 |
| 2 (6) | threonyl-tRNA synthetase | ZP_02734724 | 70497 | 387 | 12 | 13.1 |
| 2 (3,6) | negative regulator of genetic competence ClpC/MecB | ZP_02733990 | 94501 | 870 | 76 | 20.9 |
| 2 (6) | hypothetical protein GobsU_30765 | ZP_02736233 | 70604 | 199 | 9 | 7.5 |
| 2 (3) | hypothetical protein GobsU_38353 | ZP_02737734 | 62324 | 223 | 10 | 10.5 |
| 2 | hypothetical protein GobsU_07582 | ZP_02731641 | 70264 | 237 | 12 | 11.2 |
| 2 | hypothetical protein GobsU_35775 | ZP_02737224 | 79841 | 274 | 11 | 8.9 |
| 2 (3,6) | elongation factor G | ZP_02735069 | 63641 | 276 | 9 | 10.3 |
| 2 | hypothetical protein GobsU_17665 | ZP_02733634 | 75700 | 295 | 11 | 8.3 |
| 2 | probable chemotaxis sensory transducer | ZP_02731334 | 64063 | 296 | 7 | 12 |
| 2 (3) | Na-Ca exchanger/integrin-beta4 | ZP_02733245 | 73354 | 579 | 20 | 24.3 |
| 2 (6) | GTP-binding elongation factor | ZP_02732217 | 67411 | 600 | 34 | 19.7 |
| 2 | hypothetical protein GobsU_25226 | ZP_02735133 | 29992 | 600 | 21 | 30.7 |
| 2 | putative multi-domain protein | ZP_02734623 | 28378 | 544 | 65 | 39.4 |
| 2 (3) | 50S ribosomal protein L25/general stress protein Ctc | ZP_02736396 | 23172 | 504 | 38 | 42.6 |
| 2 | hypothetical protein GobsU_17625 | ZP_02733626 | 26218 | 493 | 28 | 48 |
| 2 (6) | hypothetical protein GobsU_26266 | ZP_02735339 | 27891 | 453 | 23 | 23 |
| 2 (3) | hypothetical protein GobsU_27941 | ZP_02735673 | 28277 | 413 | 15 | 26.9 |
| 2 | hypothetical protein GobsU_36335 | ZP_02737336 | 32111 | 368 | 9 | 23.8 |
| 2 | D-mannonate oxidoreductase | ZP_02732427 | 27806 | 354 | 21 | 23 |
| 2 | hypothetical cytosolic protein | ZP_02733142 | 27103 | 353 | 15 | 25.2 |
| 2 (6) | hypothetical protein GobsU_30964 | ZP_02736272 | 23254 | 311 | 11 | 29.5 |
| 2 (3,6) | 30S ribosomal protein S4 | ZP_02736977 | 22489 | 514 | 77 | 38.8 |
| 2 | hypothetical protein GobsU_37972 | ZP_02737659 | 31736 | 281 | 16 | 18.7 |
| 2 | ABC transporter ATP-binding protein | ZP_02732495 | 26694 | 271 | 7 | 22.2 |
| 2 | ABC transporter ATP-binding protein | ZP_02731083 | 71649 | 241 | 9 | 7.8 |
| 2 | short-chain dehydrogenase/reductase SDR | ZP_02732138 | 23811 | 143 | 7 | 11.9 |
| 2 | short-chain dehydrogenase/reductase SDR | ZP_02736353 | 27181 | 266 | 7 | 18.1 |
| 2 | short-chain dehydrogenase/reductase SDR | ZP_02730741 | 30327 | 217 | 4 | 19.5 |
| 2 (3,6) | hypothetical protein GobsU_25221 | ZP_02735132 | 31969 | 337 | 34 | 24.6 |
| 2 | hypothetical protein GobsU_35950 | ZP_02737259 | 31056 | 252 | 8 | 20.6 |
| 2 | SSU ribosomal protein S9P | ZP_02737482 | 22532 | 244 | 24 | 29 |
| 2 | Sulfate/thiosulfate import ATP-binding protein cysA | ZP_02731051 | 27037 | 236 | 10 | 26.7 |
| 2 | dihydrodipicolinate reductase | ZP_02734700 | 28100 | 233 | 8 | 13.7 |
| 2 | tryptophan synthase alpha chain | ZP_02731237 | 28505 | 265 | 10 | 23 |
| 2 | NIPSNAP family containing protein | ZP_02732973 | 28688 | 286 | 14 | 21.7 |
| 2 | ribosomal protein L9 | ZP_02736392 | 20902 | 342 | 16 | 28.1 |
| 2 (6) | hypothetical protein GobsU_11080 | ZP_02732339 | 35572 | 384 | 14 | 25.5 |
| 2 | hypothetical protein GobsU_09481 | ZP_02732020 | 34215 | 215 | 7 | 17.9 |
| 2 (3,6) | hypothetical protein GobsU_24726 | ZP_02735033 | 29233 | 568 | 27 | 33.7 |
| 2 | hypothetical protein GobsU_28900 | ZP_02735864 | 29227 | 125 | 5 | 16.7 |
| 2 | hypothetical protein GobsU_13877 | ZP_02732890 | 35419 | 313 | 34 | 23.3 |
| 2 | probable phosphoesterase | ZP_02734400 | 28951 | 118 | 4 | 18.5 |
| 2 | hypothetical protein GobsU_10698 | ZP_02732263 | 27768 | 115 | 8 | 14.7 |
| 2 | phosphoesterase, PA-phosphatase related protein | ZP_02730579 | 58312 | 682 | 28 | 26.7 |
| 2 (3,6) | hypothetical protein GobsU_29154 | ZP_02735914 | 33558 | 380 | 21 | 25.2 |
| 2 | hypothetical protein GobsU_02788 | ZP_02730695 | 33058 | 305 | 15 | 26.1 |
| 2 (3,6) | hypothetical protein GobsU_08407 | ZP_02731806 | 36568 | 145 | 10 | 10.2 |
| 2 | hypothetical protein GobsU_27841 | ZP_02735653 | 63110 | 305 | 8 | 12.2 |
| 2 | hypothetical protein GobsU_29553 | ZP_02735993 | 26987 | 119 | 4 | 15.6 |
| 2 | 5-formyltetrahydrofolate cyclo-ligase | ZP_02732856 | 23328 | 115 | 3 | 14.5 |
| 2 | 3-ketoacyl-(acyl-carrier-protein) reductase | ZP_02735123 | 25082 | 156 | 4 | 11.3 |
| 2 | translation initiation factor IF-3 | ZP_02734237 | 22323 | 153 | 11 | 16.7 |
| 2 | hypothetical protein GobsU_03729 | ZP_02730882 | 20378 | 206 | 5 | 24.9 |
| 2 | hypothetical protein GobsU_01552 | ZP_02730453 | 31476 | 198 | 7 | 22.1 |
| 2 | Protein kinase:GAF | ZP_02730508 | 73025 | 316 | 6 | 8.1 |
| 2 | peptidase S9, prolyl oligopeptidase active site domain protein | ZP_02736956 | 110508 | 902 | 38 | 34 |
| 2 | hypothetical protein GobsU_17520 | ZP_02733605 | 32828 | 298 | 9 | 19.2 |
| 2 | acetylglutamate kinase | ZP_02735209 | 31245 | 206 | 6 | 16.6 |
| 2 | hypothetical protein GobsU_22402 | ZP_02734571 | 25373 | 312 | 13 | 24.7 |
| 2 | 4Fe-4S ferredoxin iron-sulfur binding domain protein | ZP_02735878 | 27903 | 3217 | 479 | 48.3 |
| 2 (6) | chaperone protein HtpG | ZP_02733601 | 27919 | 176 | 17 | 13.1 |
| 2 (6) | ABC transporter (glutamine transport ATP-binding protein) | ZP_02735279 | 25792 | 160 | 5 | 21.7 |
| 2 (3) | ribosomal protein L17 | ZP_02733782 | 20725 | 290 | 19 | 21.8 |
| 2 (3,6) | 30S ribosomal protein S3 | ZP_02734657 | 28381 | 107 | 3 | 16 |
| 2 | acetolactate synthase III | ZP_02737021 | 64739 | 428 | 16 | 20.5 |
| 2 (3,6) | probable serine/threonine protein kinase related protein | ZP_02734134 | 84113 | 872 | 27 | 24.1 |
| 2 (3) | flagellar basal body rod protein | ZP_02731971 | 25399 | 107 | 4 | 18.5 |
| 2 | ABC transporter, ATPase subunit | ZP_02733332 | 27639 | 436 | 10 | 24.3 |
| 2 (3) | hypothetical protein GobsU_27311 | ZP_02735547 | 39432 | 209 | 6 | 12.2 |
| 2 | glucose-1-phosphate thymidylyltransferase (strD) | ZP_02737087 | 24780 | 177 | 7 | 14.9 |
| 2 (6) | lipoprotein releasing system ATP-binding protein lolD | ZP_02734712 | 24120 | 168 | 5 | 21.9 |
| 2 (3) | hypothetical protein GobsU_26961 | ZP_02735478 | 20743 | 303 | 10 | 26.4 |
| 2 | thioredoxin peroxidase | ZP_02735335 | 22012 | 310 | 27 | 26.3 |
| 2 (3) | hypothetical protein GobsU_17515 | ZP_02733604 | 21816 | 255 | 13 | 33.7 |
| 2 (3) | hypothetical protein GobsU_03025 | ZP_02730742 | 21233 | 235 | 27 | 23.6 |
| 2 (3) | nitroreductase | ZP_02737361 | 23271 | 636 | 29 | 42.3 |
| 2 | LexA repressor | ZP_02737915 | 26890 | 209 | 7 | 18.9 |
| 2 | acetyl-CoA carboxylase (biotin carboxyl carrier subunit) accB | ZP_02731261 | 18550 | 170 | 5 | 25.9 |
| 2 | ATP--cobalamin adenosyltransferase | ZP_02736319 | 21124 | 136 | 4 | 14.6 |
| 2 | Dipeptidyl aminopeptidase | ZP_02736772 | 83036 | 522 | 20 | 14.5 |
| 2 | ribonuclease E | ZP_02731873 | 108200 | 590 | 35 | 14.4 |
| 2 | hypothetical protein GobsU_29289 | ZP_02735941 | 68122 | 708 | 22 | 24.7 |
| 2 | bifunctional sulfate adenylyltransferase subunit 1/adenylylsulfate kinase protein | ZP_02733159 | 70580 | 172 | 6 | 10.7 |
| 2 | putative signal transduction protein | ZP_02734853 | 22925 | 196 | 12 | 37.6 |
| 2 | multi-sensor hybrid histidine kinase | ZP_02734427 | 108654 | 244 | 6 | 3.9 |
| 2 | multi-sensor hybrid histidine kinase | ZP_02732316 | 68813 | 299 | 8 | 14.5 |
| 2 | multi-sensor hybrid histidine kinase | ZP_02731339 | 72445 | 250 | 8 | 8.4 |
| 2 | multi-sensor hybrid histidine kinase | ZP_02737274 | 39793 | 204 | 6 | 14.8 |
| 2 | probable sensor kinase | ZP_02735899 | 61961 | 374 | 14 | 18.9 |
| 2 | phosphomannomutase | ZP_02732506 | 71596 | 305 | 13 | 16.9 |
| 2 (3) | hypothetical protein GobsU_29361 | ZP_02735955 | 91983 | 738 | 43 | 19.5 |
| 2 | 2-oxoglutarate ferredoxin oxidoreductase alpha subunit | ZP_02733920 | 69353 | 680 | 37 | 25.7 |
| 2 | hypothetical protein GobsU_12802 | ZP_02732679 | 78483 | 626 | 35 | 19.9 |
| 2 (6) | probable secreted glycosyl hydrolase | ZP_02734952 | 155793 | 844 | 26 | 11.6 |
| 2 | transcription initiation factor sigma 70 | ZP_02737037 | 64210 | 386 | 11 | 17.1 |
| 2 | hypothetical protein GobsU_11425 | ZP_02732408 | 74604 | 533 | 31 | 16.5 |
| 2 | probable adenylate cyclase | ZP_02735779 | 70674 | 498 | 17 | 17.8 |
| 2 | hypothetical protein GobsU_06118 | ZP_02731353 | 75215 | 476 | 13 | 18.1 |
| 2 | heat shock protein 90 | ZP_02737391 | 69763 | 456 | 18 | 16.2 |
| 2 | signal transduction histidine kinase with CheB and CheR activity | ZP_02733916 | 66728 | 333 | 8 | 13.3 |
| 2 | hypothetical protein GobsU_38503 | ZP_02737764 | 68151 | 290 | 8 | 10.6 |
| 2 | DNA gyrase subunit B | ZP_02732070 | 71463 | 275 | 15 | 14 |
| 2 | translation initiation factor IF-2 | ZP_02736305 | 124152 | 270 | 9 | 5.9 |
| 2 | D-galactarate dehydratase/altronate hydrolase-like protein | ZP_02735035 | 55918 | 399 | 12 | 17.6 |
| 2 | hypothetical protein GobsU_36582 | ZP_02737385 | 59802 | 372 | 8 | 11.9 |
| 2 | hypothetical protein GobsU_30335 | ZP_02736147 | 58651 | 308 | 9 | 17.5 |
| 2 | thiamine-phosphate pyrophosphorylase | ZP_02732066 | 52913 | 373 | 13 | 23.5 |
| 2 | hypothetical protein GobsU_06705 | ZP_02731470 | 57820 | 199 | 7 | 11 |
| 2 | phosphoglycerate dehydrogenase | ZP_02733260 | 56312 | 371 | 11 | 13.2 |
| 2 | two-component system sensory histidine kinase | ZP_02737190 | 58308 | 202 | 4 | 9.5 |
| 2 | cetoacetate metabolism regulatory protein atoC | ZP_02733210 | 52917 | 328 | 10 | 15.1 |
| 2 | cyanophycinase | ZP_02733322 | 57350 | 208 | 5 | 8.7 |
| 2 (3) | hypothetical protein GobsU_09903 | ZP_02732104 | 49991 | 307 | 7 | 13.8 |
| 2 | histidinol dehydrogenase | ZP_02736566 | 51372 | 292 | 8 | 19 |
| 2 (6) | probable auxin-responsive-like protein | ZP_02733619 | 63167 | 216 | 6 | 9.6 |
| 2 | Amidase | ZP_02736331 | 59678 | 591 | 9 | 13 |
| 2 (3,6) | hypothetical protein GobsU_14664 | ZP_02733041 | 37661 | 426 | 32 | 19.9 |
| 2 (3) | hypothetical protein GobsU_23747 | ZP_02734840 | 47608 | 761 | 25 | 23 |
| 2 (3) | hypothetical protein GobsU_34987 | ZP_02737073 | 41782 | 652 | 20 | 18 |
| 2 (3) | Collagen triple helix repeat | ZP_02737845 | 43510 | 329 | 9 | 6 |
| 2 (3) | hypothetical protein GobsU_33449 | ZP_02736767 | 25070 | 98 | 3 | 13.6 |
| 2 (3) | hypothetical protein GobsU_39193 | ZP_02737902 | 37851 | 226 | 7 | 15.4 |
| 2 (3,6) | hypothetical protein GobsU_20648 | ZP_02734225 | 74282 | 309 | 11 | 10.3 |
| 2 (3) | hypothetical protein GobsU_28690 | ZP_02735822 | 38321 | 316 | 8 | 14.8 |
| 2 (6) | hypothetical protein GobsU_29901 | ZP_02736061 | 51041 | 287 | 6 | 6 |
| 2 (3) | probable tolQ protein | ZP_02733303 | 29569 | 171 | 12 | 12.3 |
| 2 | protein translation elongation factor P (EF-P) | ZP_02732409 | 21479 | 188 | 17 | 23.6 |
| 2 | sigma-24, ECF subfamily protein | ZP_02735550 | 19550 | 109 | 4 | 16.8 |
| 2 (3) | hypothetical protein GobsU_11650 | ZP_02732451 | 35030 | 655 | 40 | 31.8 |
| 2 (3) | hypothetical protein GobsU_25326 | ZP_02735153 | 23124 | 200 | 5 | 10.9 |
| 2 (6) | efflux transporter, RND family, MFP subunit | ZP_02732378 | 48939 | 226 | 8 | 10.7 |
| 2 (3) | hypothetical protein GobsU_34792 | ZP_02737034 | 161460 | 595 | 20 | 16 |
| 2 (6) | DNA-directed RNA polymerase subunit alpha | ZP_02733781 | 37755 | 914 | 45 | 32.7 |
| 2 (6) | twitching mobility protein PilT | ZP_02734150 | 43895 | 513 | 13 | 23.5 |
| 2 (6) | twitching motility protein PilT | ZP_02733546 | 43722 | 353 | 8 | 13.9 |
| 2 (6) | hypothetical protein GobsU_26261 | ZP_02735338 | 45236 | 207 | 6 | 9.2 |
| 2 | probable NADH-dependent dehydrogenase | ZP_02734856 | 51921 | 194 | 6 | 7.9 |
| 2 (3) | hypothetical protein GobsU_16177 | ZP_02733342 | 43879 | 548 | 15 | 31.5 |
| 2 (6) | Aldose 1-epimerase | ZP_02734020 | 41900 | 303 | 8 | 13.2 |
| 2 (6) | hypothetical protein GobsU_20243 | ZP_02734144 | 41028 | 386 | 15 | 18.3 |
| 2 (3) | hypothetical protein GobsU_17361 | ZP_02733574 | 145135 | 553 | 23 | 6.6 |
| 2 | hypothetical protein GobsU_27771 | ZP_02735639 | 36936 | 435 | 16 | 22.2 |
| 2 (3) | polysaccharide export protein | ZP_02736601 | 40175 | 346 | 15 | 17.5 |
| 2 (3) | hypothetical protein GobsU_32114 | ZP_02736502 | 41942 | 238 | 9 | 10 |
| 2 | hypothetical protein GobsU_17136 | ZP_02733529 | 31873 | 222 | 7 | 13 |
| 2 | GDP-mannose 4,6-dehydratase | ZP_02734112 | 37597 | 221 | 14 | 13.9 |
| 2 (6) | cytochrome c oxidase, subunit II | ZP_02733609 | 41126 | 816 | 36 | 36 |
| 2 (3,6) | hypothetical protein GobsU_12210 | ZP_02732563 | 41758 | 104 | 3 | 6.2 |
| 2 (6) | hypothetical protein GobsU_14991 | ZP_02733106 | 39437 | 639 | 18 | 33 |
| 2 (6) | hypothetical protein GobsU_20238 | ZP_02734143 | 37132 | 413 | 21 | 23.1 |
| 2 | hypothetical protein GobsU_30685 | ZP_02736217 | 36258 | 288 | 7 | 21.1 |
| 2 (3) | hypothetical protein GobsU_25386 | ZP_02735165 | 44361 | 272 | 8 | 10.2 |
| 2 (6) | PfkB domain protein | ZP_02737066 | 39152 | 260 | 7 | 12.7 |
| 2 (3,6) | 30S ribosomal protein S2 | ZP_02737376 | 26492 | 310 | 10 | 30.4 |
| 2 | hypothetical protein GobsU_30580 | ZP_02736196 | 36997 | 145 | 4 | 6.9 |
| 2 (6) | hypothetical protein GobsU_17530 | ZP_02733607 | 26219 | 339 | 20 | 25.9 |
| 2 | hypothetical protein GobsU_11145 | ZP_02732352 | 39951 | 324 | 18 | 8.7 |
| 2 | hypothetical protein GobsU_31734 | ZP_02736426 | 37527 | 237 | 23 | 14.5 |
| 2 (6) | hypothetical protein GobsU_32284 | ZP_02736536 | 34391 | 216 | 8 | 15.4 |
| 2 (6) | hypothetical protein GobsU_36654 | ZP_02737399 | 34611 | 614 | 31 | 43 |
| 2 (3) | hypothetical protein GobsU_14649 | ZP_02733038 | 36583 | 475 | 19 | 29.3 |
| 2 (6) | hypothetical protein GobsU_30670 | ZP_02736214 | 35019 | 463 | 17 | 33.1 |
| 2 (3,6) | hypothetical protein GobsU_11730 | ZP_02732467 | 38243 | 428 | 27 | 30.2 |
| 2 | hypothetical protein GobsU_16057 | ZP_02733318 | 33108 | 314 | 13 | 21.1 |
| 2 (3) | hypothetical protein GobsU_05341 | ZP_02731198 | 34681 | 278 | 8 | 23 |
| 2 (3,6) | hypothetical protein GobsU_26271 | ZP_02735340 | 33045 | 237 | 9 | 14.5 |
| 2 | hypothetical protein GobsU_25211 | ZP_02735130 | 31614 | 123 | 3 | 11.6 |
| 2 (3,6) | hypothetical protein GobsU_20248 | ZP_02734145 | 35110 | 143 | 5 | 9.7 |
| 2 | hypothetical protein GobsU_18200 | ZP_02733741 | 34127 | 336 | 11 | 15.5 |
| 2 | hypothetical protein GobsU_36425 | ZP_02737354 | 31866 | 241 | 6 | 18 |
| 2 | hypothetical protein GobsU_38508 | ZP_02737765 | 34806 | 121 | 3 | 7.5 |
| 2 (3) | hypothetical protein GobsU_37727 | ZP_02737610 | 34268 | 195 | 8 | 11.5 |
| 2 (3) | hypothetical protein GobsU_21390 | ZP_02734373 | 28539 | 167 | 3 | 15.7 |
| 2 | hypothetical protein GobsU_31319 | ZP_02736343 | 30456 | 112 | 3 | 11.9 |
| **Summary for Fraction 2** | **119 proteins are unique for this fraction (in green) – 44 % of total** | **34 proteins overlap with proteins from fractions 3 and 6** | **36 proteins overlap with proteins from fraction 3** | **82 proteins overlap with proteins from fraction 6** |  | **271 proteins in total** |
| **Fraction 3 (Pore-containing membrane fraction;** if protein found in other fractions it is indicated in brackets) | **Protein name** | **NCBI accession numbers** | **Calculated molecular weight (Da)*** | **MOWSE score** | **Peptides matched** | **Sequence coverage (%)** |
| 3 (2,6) | aconitate hydratase 1 | ZP_02730459 | 98013 | 767 | 26 | 23.1 |
| 3 (2) | hypothetical protein GobsU_16177 | ZP_02733342 | 43879 | 651 | 40 | 35.5 |
| 3 (2,6) | 30S ribosomal protein S3 | ZP_02734657 | 28381 | 250 | 3 | 19.8 |
| 3 (6) | 50S ribosomal protein L5 | ZP_02734651 | 20975 | 158 | 11 | 18 |
| 3 (2,6) | hypothetical protein GobsU_27236 | ZP_02735532 | 35749 | 662 | 31 | 36.3 |
| 3 (2,6) | hypothetical protein GobsU_20248 | ZP_02734145 | 35110 | 359 | 5 | 25.9 |
| 3 (2,6) | hypothetical protein GobsU_14664 | ZP_02733041 | 37661 | 313 | 6 | 16.5 |
| 3 (2,6) | hypothetical protein GobsU_24726 | ZP_02735033 | 29233 | 310 | 5 | 19.3 |
| 3 (2,6) | hypothetical protein GobsU_29154 | ZP_02735914 | 33558 | 297 | 3 | 17.9 |
| 3 (2) | hypothetical protein GobsU_11650 | ZP_02732451 | 35030 | 414 | 19 | 20.8 |
| 3 (6) | hypothetical protein GobsU_10668 | ZP_02732257 | 29414 | 569 | 45 | 32.4 |
| 3 (2,6) | hypothetical protein GobsU_11730 | ZP_02732467 | 38243 | 243 | 4 | 23 |
| 3 (2,6) | hypothetical protein GobsU_08407 | ZP_02731806 | 36568 | 225 | 3 | 12.8 |
| 3 (6) | 50S ribosomal protein L4 | ZP_02734662 | 25590 | 210 | 3 | 17 |
| 3 (2) | hypothetical protein GobsU_37727 | ZP_02737610 | 34268 | 178 | 3 | 11.1 |
| 3 (2) | hypothetical protein GobsU_14649 | ZP_02733038 | 36583 | 166 | 3 | 11 |
| 3 (2) | hypothetical protein GobsU_05341 | ZP_02731198 | 34681 | 160 | 3 | 16.6 |
| 3 (2,6) | 50S ribosomal protein L1 | ZP_02733084 | 30488 | 169 | 12 | 10 |
| 3 (2) | hypothetical protein GobsU_38353 | ZP_02737734 | 62324 | 382 | 8 | 19.4 |
| 3 (2,6) | hypothetical protein GobsU_38668 | ZP_02737797 | 58509 | 860 | 74 | 26.5 |
| 3 (2) | hypothetical protein GobsU_25326 | ZP_02735153 | 23124 | 332 | 18 | 23.5 |
| 3 (2,6) | 30S ribosomal protein S4 | ZP_02736977 | 22489 | 472 | 25 | 28.6 |
| 3 | hypothetical protein GobsU_26936 | ZP_02735473 | 32285 | 524 | 7 | 36.1 |
| 3 | probable polysaccharide export protein | ZP_02733516 | 30207 | 264 | 5 | 32.9 |
| 3 (2) | polysaccharide export protein | ZP_02736601 | 40175 | 549 | 24 | 30.1 |
| 3 (2) | hypothetical protein GobsU_25386 | ZP_02735165 | 44361 | 697 | 10 | 33.6 |
| 3 (2,6) | 30S ribosomal protein S2 | ZP_02737376 | 26492 | 152 | 4 | 23.8 |
| 3 (6) | putative small-conductance mechanosensitive ion channel | ZP_02737296 | 96665 | 372 | 10 | 11.3 |
| 3 (2) | hypothetical protein GobsU_23747 | ZP_02734840 | 47608 | 828 | 47 | 30 |
| 3 | hypothetical protein GobsU_28460 | ZP_02735776 | 43365 | 492 | 24 | 24.7 |
| 3 | hypothetical protein GobsU_27906 | ZP_02735666 | 43560 | 334 | 4 | 17.4 |
| 3 (2) | hypothetical protein GobsU_34987 | ZP_02737073 | 41782 | 995 | 145 | 49.6 |
| 3 | Na-Ca exchanger/integrin-beta4 | ZP_02736670 | 43599 | 374 | 6 | 15.4 |
| 3 | hypothetical protein GobsU_12560 | ZP_02732631 | 49640 | 650 | 37 | 26.1 |
| 3 | hypothetical protein GobsU_03779 | ZP_02730892 | 39742 | 495 | 22 | 24.9 |
| 3 | hypothetical protein GobsU_05311 | ZP_02731192 | 42925 | 559 | 9 | 27 |
| 3 (2) | hypothetical protein GobsU_29361 | ZP_02735955 | 91983 | 921 | 47 | 26.3 |
| 3 (2,6) | negative regulator of genetic competence ClpC/MecB | ZP_02733990 | 94501 | 893 | 36 | 25.8 |
| 3 | hypothetical protein GobsU_06270 | ZP_02731383 | 94980 | 1312 | 55 | 35.1 |
| 3 | hypothetical protein GobsU_23427 | ZP_02734776 | 96132 | 605 | 19 | 14.1 |
| 3 | hypothetical protein GobsU_27926 | ZP_02735670 | 21683 | 296 | 10 | 25.5 |
| 3 (6) | Scramblase family protein | ZP_02730790 | 21656 | 250 | 5 | 32.5 |
| 3 (2) | hypothetical protein GobsU_17515 | ZP_02733604 | 21816 | 216 | 10 | 29.6 |
| 3 (2) | hypothetical protein GobsU_03025 | ZP_02730742 | 21233 | 138 | 6 | 18.6 |
| 3 | hypothetical protein GobsU_32159 | ZP_02736511 | 106755 | 2423 | 182 | 42.7 |
| 3 | hypothetical protein GobsU_17066 | ZP_02733515 | 138451 | 896 | 25 | 16.4 |
| 3 | hypothetical protein GobsU_09718 | ZP_02732067 | 50348 | 660 | 17 | 25.8 |
| 3 | probable divalent cation resistant determinant protein C | ZP_02731891 | 46316 | 471 | 11 | 19.5 |
| 3 (2,6) | oxidoreductase domain protein | ZP_02735697 | 47995 | 583 | 14 | 23.9 |
| 3 (2,6) | translation-associated GTPase | ZP_02730857 | 39663 | 417 | 19 | 19.6 |
| 3 (2) | Collagen triple helix repeat | ZP_02737845 | 43510 | 349 | 10 | 17.1 |
| 3 (2,6) | putative peptidase | ZP_02736310 | 42703 | 313 | 11 | 18.5 |
| 3 | hypothetical protein GobsU_29613 | ZP_02736005 | 41551 | 238 | 5 | 11.2 |
| 3 (2) | hypothetical protein GobsU_27941 | ZP_02735673 | 28277 | 611 | 24 | 41.3 |
| 3 (2) | flagellar basal body rod protein FlgG | ZP_02731970 | 27437 | 495 | 29 | 29.7 |
| 3 (2) | flagellar basal body rod protein | ZP_02731971 | 25399 | 182 | 4 | 18.5 |
| 3 (2) | 50S ribosomal protein L25/general stress protein Ctc | ZP_02736396 | 23172 | 262 | 8 | 24.5 |
| 3 (2,6) | hypothetical protein GobsU_20253 | ZP_02734146 | 26677 | 233 | 9 | 16.2 |
| 3 (2,6) | hypothetical protein GobsU_25221 | ZP_02735132 | 31969 | 157 | 8 | 12.6 |
| 3 (2) | hypothetical protein GobsU_32114 | ZP_02736502 | 41942 | 638 | 33 | 32.5 |
| 3 | hypothetical protein GobsU_33214 | ZP_02736720 | 43019 | 186 | 6 | 11.8 |
| 3 (6) | hypothetical protein GobsU_01252 | ZP_02730395 | 38353 | 198 | 6 | 14.7 |
| 3 (6) | hypothetical protein GobsU_20093 | ZP_02734114 | 28967 | 213 | 6 | 17.3 |
| 3 (2) | hypothetical protein GobsU_26961 | ZP_02735478 | 20743 | 356 | 12 | 34.2 |
| 3 (2) | nitroreductase | ZP_02737361 | 23271 | 307 | 8 | 31.2 |
| 3 (2) | hypothetical protein GobsU_33449 | ZP_02736767 | 25070 | 115 | 4 | 13.6 |
| 3 | hypothetical protein GobsU_36674 | ZP_02737403 | 13718 | 108 | 4 | 10.4 |
| 3 | hypothetical protein GobsU_22432 | ZP_02734577 | 92982 | 788 | 82 | 16.3 |
| 3 | hypothetical protein GobsU_18530 | ZP_02733807 | 114144 | 566 | 36 | 13.8 |
| 3 | autotransporter-associated beta strand repeat protein | ZP_02735782 | 111523 | 983 | 150 | 15.2 |
| 3 (2,6) | hypothetical protein GobsU_06435 | ZP_02731416 | 103608 | 428 | 15 | 13.1 |
| 3 (6) | hypothetical protein GobsU_09596 | ZP_02732043 | 81831 | 378 | 19 | 13.7 |
| 3 (2) | hypothetical protein GobsU_27311 | ZP_02735547 | 39432 | 286 | 8 | 12.7 |
| 3 (6) | hypothetical protein GobsU_14884 | ZP_02733085 | 19370 | 237 | 7 | 19.7 |
| 3 (2) | hypothetical protein GobsU_39193 | ZP_02737902 | 37851 | 196 | 19 | 10.4 |
| 3 (6) | hypothetical protein GobsU_20228 | ZP_02734141 | 177838 | 1152 | 38 | 18.9 |
| 3 | hypothetical protein GobsU_01822 | ZP_02730507 | 122792 | 1726 | 87 | 29.9 |
| 3 | hypothetical protein GobsU_02172 | ZP_02730573 | 81027 | 1136 | 55 | 32.4 |
| 3 | hypothetical protein GobsU_28980 | ZP_02735880 | 102196 | 1106 | 42 | 21.8 |
| 3 (2,6) | hypothetical protein GobsU_20643 | ZP_02734224 | 92712 | 1032 | 48 | 26.2 |
| 3 (2,6) | hypothetical protein GobsU_31654 | ZP_02736410 | 87810 | 939 | 33 | 23 |
| 3 (2) | Na-Ca exchanger/integrin-beta4 | ZP_02733245 | 73354 | 813 | 23 | 29.5 |
| 3 | peroxidase/catalase | ZP_02730801 | 86701 | 786 | 25 | 22.7 |
| 3 (2,6) | probable serine/threonine protein kinase related protein | ZP_02734134 | 84113 | 694 | 42 | 22.3 |
| 3 (6) | probable fimbrial assembly protein PilM | ZP_02734226 | 88149 | 684 | 23 | 19 |
| 3 (2,6) | hypothetical protein GobsU_29906 | ZP_02736062 | 76943 | 609 | 17 | 24.7 |
| 3 (2,6) | hypothetical protein GobsU_20648 | ZP_02734225 | 74282 | 591 | 20 | 19.2 |
| 3 | hypothetical protein GobsU_22867 | ZP_02734664 | 90756 | 422 | 12 | 17.3 |
| 3 (6) | polynucleotide phosphorylase/polyadenylase | ZP_02731046 | 80887 | 402 | 16 | 15.8 |
| 3 (2) | hypothetical protein GobsU_17361 | ZP_02733574 | 145135 | 862 | 58 | 10.1 |
| 3 (6) | hypothetical protein GobsU_34877 | ZP_02737051 | 82715 | 326 | 9 | 10.2 |
| 3 | hypothetical protein GobsU_21510 | ZP_02734397 | 37960 | 583 | 17 | 29.5 |
| 3 | hypothetical protein GobsU_04229 | ZP_02730982 | 28031 | 404 | 14 | 32.6 |
| 3 (2) | hypothetical protein GobsU_21390 | ZP_02734373 | 28539 | 435 | 10 | 33.6 |
| 3 (2) | hypothetical protein GobsU_28690 | ZP_02735822 | 38321 | 350 | 22 | 18.4 |
| 3 (6) | hypothetical protein GobsU_19324 | ZP_02733961 | 107113 | 527 | 16 | 13.7 |
| 3 (2) | probable tolQ protein | ZP_02733303 | 29569 | 175 | 5 | 12.3 |
| 3 | hypothetical protein GobsU_23637 | ZP_02734818 | 254017 | 1257 | 74 | 7.9 |
| 3 | FG-GAP repeat protein | ZP_02731030 | 163956 | 668 | 23 | 8.4 |
| 3 | peptidase S8 and S53, subtilisin, kexin, sedolisin | ZP_02737072 | 64444 | 432 | 7 | 13.6 |
| 3 (2,6) | hypothetical protein GobsU_12210 | ZP_02732563 | 41758 | 229 | 7 | 11.7 |
| 3 (6) | flagellar motor switch protein G | ZP_02730986 | 32972 | 459 | 16 | 31.8 |
| 3 (2) | type IV fimbrial assembly protein PilB | ZP_02734149 | 70569 | 573 | 17 | 15.8 |
| 3 (2,6) | 30S ribosomal protein S1 | ZP_02736868 | 69112 | 191 | 7 | 8.3 |
| 3 (6) | 50S ribosomal protein L3 | ZP_02737246 | 32224 | 113 | 2 | 8.6 |
| 3 (2,6) | glutamine synthetase, catalytic region | ZP_02737578 | 79979 | 581 | 16 | 17.6 |
| 3 (2,6) | hypothetical protein GobsU_20233 | ZP_02734142 | 48877 | 296 | 10 | 17.5 |
| 3 (2,6) | elongation factor G | ZP_02735069 | 63641 | 337 | 10 | 13 |
| 3 (2) | Uridylate kinase | ZP_02736756 | 26457 | 149 | 4 | 12 |
| 3 | hypothetical protein GobsU_02177 | ZP_02730574 | 73578 | 561 | 25 | 23.3 |
| 3 (2) | ribosomal protein L17 | ZP_02733782 | 20725 | 217 | 3 | 11.7 |
| 3 (2) | hypothetical protein GobsU_34792 | ZP_02737034 | 161460 | 1209 | 155 | 11.1 |
| 3 (2,6) | hypothetical protein GobsU_04904 | ZP_02731113 | 61669 | 798 | 34 | 28.5 |
| 3 | probable outer membrane lipoprotein IbeB | ZP_02736193 | 58744 | 1021 | 86 | 27.7 |
| 3 (2,6) | hypothetical protein GobsU_38147 | ZP_02737694 | 46334 | 280 | 26 | 14 |
| 3 | hypothetical protein GobsU_31139 | ZP_02736307 | 52674 | 195 | 5 | 16.9 |
| 3 (2,6) | Flagellar FliF M-ring protein | ZP_02730987 | 54974 | 329 | 9 | 16.4 |
| 3 (2) | hypothetical protein GobsU_28805 | ZP_02735845 | 67828 | 1291 | 122 | 38.2 |
| 3 (2) | hypothetical protein GobsU_09903 | ZP_02732104 | 49991 | 817 | 29 | 32.4 |
| 3 | hypothetical protein GobsU_13562 | ZP_02732829 | 57915 | 595 | 45 | 20.9 |
| 3 (6) | 30S ribosomal protein S7 | ZP_02733090 | 17959 | 462 | 15 | 44.3 |
| 3 (6) | 30S ribosomal protein S8 | ZP_02734649 | 17113 | 334 | 9 | 52.6 |
| 3 | UspA domain protein | ZP_02737803 | 13406 | 183 | 3 | 19.8 |
| 3 | hypothetical protein GobsU_05481 | ZP_02731226 | 17416 | 174 | 4 | 19.1 |
| 3 (6) | ribosomal protein L21 | ZP_02733065 | 13902 | 134 | 4 | 24.6 |
| 3 | hypothetical protein GobsU_31204 | ZP_02736320 | 21793 | 96 | 4 | 15 |
| 3 (2,6) | hypothetical protein GobsU_26271 | ZP_02735340 | 33045 | 361 | 13 | 24.8 |
| 3 | hypothetical protein GobsU_09169 | ZP_02731958 | 30276 | 294 | 6 | 13.4 |
| **Summary for Fraction 3** | **39 proteins are unique for this fraction (in green)-30.5% of total** | **34 proteins overlap with proteins from fractions 2 and 6** | **36 proteins overlap with proteins from fraction 2** | **19 proteins overlap with proteins from fraction 6** |  | **128 proteins in total** |
| **Fraction 6** (if protein found in other fractions it is indicated in brackets) | **Protein name** | **NCBI accession numbers** | **Calculated molecular weight (Da)*** | **MOWSE score** | **Peptides matched** | **Sequence coverage (%)** |
| 6 (2) | ATP synthase F1, alpha subunit | ZP_02731443 | 62190 | 2083 | 265 | 54.9 |
| 6 (2) | putative ABC transporter ATP-binding protein | ZP_02736693 | 34576 | 1208 | 93 | 67.9 |
| 6 | Extracellular ligand-binding receptor | ZP_02731794 | 45810 | 1061 | 62 | 49.7 |
| 6 | NuoF2 NADH I CHAIN F | ZP_02732659 | 50133 | 763 | 25 | 35.8 |
| 6 (2,3) | hypothetical protein GobsU_27236 | ZP_02735532 | 35749 | 836 | 108 | 46.2 |
| 6 (2) | DNA-directed RNA polymerase beta chain | ZP_02733087 | 141275 | 2279 | 76 | 29.7 |
| 6 | preprotein translocase subunit SecA | ZP_02731074 | 145943 | 1616 | 49 | 23.9 |
| 6 (2) | protein-export membrane protein secD | ZP_02733003 | 123971 | 2335 | 88 | 37.4 |
| 6 | transporter, hydrophobe/amphiphile efflux-1 (HAE1) family protein | ZP_02734258 | 134298 | 1010 | 42 | 20.1 |
| 6 (2) | hypothetical protein GobsU_35960 | ZP_02737261 | 103499 | 1007 | 41 | 21.8 |
| 6 | hypothetical protein GobsU_31659 | ZP_02736411 | 80096 | 754 | 26 | 23.4 |
| 6 (2) | cytochrome c oxidase, subunit II | ZP_02733609 | 41126 | 584 | 6 | 33.2 |
| 6 | hypothetical protein GobsU_09963 | ZP_02732116 | 33154 | 270 | 8 | 14.7 |
| 6 (2) | DNA-directed RNA polymerase beta chain | ZP_02733088 | 164212 | 2279 | 76 | 29.7 |
| 6 (3) | hypothetical protein GobsU_20228 | ZP_02734141 | 177838 | 1868 | 95 | 26.9 |
| 6 | hypothetical protein GobsU_32939 | ZP_02736665 | 22754 | 121 | 4 | 13.7 |
| 6 | ATP-binding protein | ZP_02731038 | 141780 | 916 | 27 | 20 |
| 6 (2,3) | hypothetical protein GobsU_06435 | ZP_02731416 | 103608 | 1683 | 102 | 32.6 |
| 6 (3) | hypothetical protein GobsU_19324 | ZP_02733961 | 107113 | 1500 | 53 | 33.5 |
| 6 (2) | probable secreted glycosyl hydrolase | ZP_02734952 | 155793 | 1256 | 36 | 18.7 |
| 6 (3) | putative small-conductance mechanosensitive ion channel | ZP_02737296 | 96665 | 1289 | 39 | 28.8 |
| 6 (2) | 4Fe-4S ferredoxin, iron-sulfur binding domain protein | ZP_02733602 | 122387 | 3144 | 378 | 48.1 |
| 6 | cyclic nucleotide-binding domain (cNMP-BD) protein | ZP_02734325 | 103286 | 1287 | 46 | 25.5 |
| 6 (2,3) | aconitate hydratase 1 | ZP_02730459 | 98013 | 1003 | 35 | 29.3 |
| 6 | pyruvate phosphate dikinase | ZP_02732609 | 96664 | 866 | 30 | 19.1 |
| 6 | probable chaperone protein DnaK | ZP_02732821 | 102245 | 832 | 30 | 22.5 |
| 6 | hypothetical protein GobsU_04604 | ZP_02731053 | 94790 | 808 | 26 | 19.4 |
| 6 (3) | hypothetical protein GobsU_09596 | ZP_02732043 | 81831 | 472 | 13 | 13.7 |
| 6 | hypothetical protein GobsU_32934 | ZP_02736664 | 70018 | 261 | 9 | 11.7 |
| 6 (2,3) | hypothetical protein GobsU_31654 | ZP_02736410 | 87810 | 2886 | 229 | 52.7 |
| 6 | Protease | ZP_02733734 | 90452 | 1453 | 61 | 36.2 |
| 6 | hypothetical protein GobsU_36485 | ZP_02737366 | 87089 | 1147 | 36 | 32.1 |
| 6 (2,3) | negative regulator of genetic competence ClpC/MecB | ZP_02733990 | 94501 | 1019 | 37 | 22.6 |
| 6 (3) | probable fimbrial assembly protein PilM | ZP_02734226 | 88149 | 601 | 18 | 13.7 |
| 6 | acylglycerophosphoethanolamine acyltransferase | ZP_02736225 | 95762 | 471 | 13 | 13.8 |
| 6 | tetratricopeptide TPR_2 | ZP_02736723 | 78679 | 1022 | 35 | 31.3 |
| 6 (3) | hypothetical protein GobsU_34877 | ZP_02737051 | 82715 | 918 | 28 | 26.2 |
| 6 (2,3) | elongation factor G | ZP_02735069 | 63641 | 767 | 23 | 27.4 |
| 6 (2,3) | probable serine/threonine protein kinase related protein | ZP_02734134 | 84113 | 465 | 13 | 15.1 |
| 6 (3) | polynucleotide phosphorylase/polyadenylase | ZP_02731046 | 80887 | 687 | 20 | 18.4 |
| 6 | hypothetical protein GobsU_06560 | ZP_02731441 | 20832 | 538 | 80 | 37.2 |
| 6 | Thioredoxin peroxidase | ZP_02734436 | 18170 | 227 | 4 | 21.6 |
| 6 | hypothetical protein GobsU_12710 | ZP_02732661 | 14951 | 188 | 8 | 26.4 |
| 6 | 30S ribosomal protein S5 | ZP_02734646 | 18356 | 118 | 7 | 21.1 |
| 6 | hypothetical protein GobsU_05059 | ZP_02731142 | 15020 | 625 | 61 | 49 |
| 6 (3) | ribosomal protein L21 | ZP_02733065 | 13902 | 127 | 4 | 24.6 |
| 6 | Redoxin domain protein | ZP_02732745 | 19451 | 130 | 8 | 11.6 |
| 6 | heat shock protein, HSP20 family | ZP_02734270 | 16043 | 100 | 7 | 16.7 |
| 6 (3) | 30S ribosomal protein S8 | ZP_02734649 | 17113 | 388 | 35 | 42.8 |
| 6 | hypothetical protein GobsU_27776 | ZP_02735640 | 14871 | 300 | 33 | 35 |
| 6 (3) | 30S ribosomal protein S7 | ZP_02733090 | 17959 | 298 | 9 | 26.6 |
| 6 | hypothetical protein GobsU_10558 | ZP_02732235 | 17920 | 278 | 10 | 21 |
| 6 | hypothetical protein GobsU_29743 | ZP_02736031 | 19895 | 250 | 16 | 28.8 |
| 6 | hypothetical protein GobsU_29149 | ZP_02735913 | 16715 | 242 | 7 | 30.2 |
| 6 | hypothetical protein GobsU_35850 | ZP_02737239 | 15435 | 215 | 12 | 26.2 |
| 6 | UspA domain protein | ZP_02737860 | 15996 | 186 | 11 | 32 |
| 6 | 50S ribosomal protein L16 | ZP_02734656 | 17323 | 158 | 4 | 18.4 |
| 6 | NADH (or F420H2) dehydrogenase, subunit C | ZP_02732663 | 19152 | 158 | 14 | 21.6 |
| 6 | probable general stress protein 26 | ZP_02736999 | 17931 | 154 | 8 | 18.3 |
| 6 | hypothetical protein GobsU_09469 | ZP_02732018 | 18718 | 129 | 6 | 15 |
| 6 | hypothetical protein GobsU_15977 | ZP_02733302 | 17748 | 88 | 2 | 10.7 |
| 6 | hypothetical protein GobsU_24731 | ZP_02735034 | 13764 | 290 | 62 | 41 |
| 6 (3) | hypothetical protein GobsU_10668 | ZP_02732257 | 29414 | 321 | 25 | 26.3 |
| 6 (2,3) | hypothetical protein GobsU_14664 | ZP_02733041 | 37661 | 299 | 8 | 14.8 |
| 6 (2,3) | hypothetical protein GobsU_20248 | ZP_02734145 | 35110 | 215 | 5 | 13.1 |
| 6 (2) | oxidoreductase, short-chain dehydrogenase/reductase family protein | ZP_02733345 | 24580 | 405 | 13 | 26.6 |
| 6 (2,3) | 50S ribosomal protein L1 | ZP_02733084 | 30488 | 215 | 9 | 14.2 |
| 6 (2) | hypothetical protein GobsU_30670 | ZP_02736214 | 35019 | 460 | 38 | 26.7 |
| 6 (2,3) | hypothetical protein GobsU_08407 | ZP_02731806 | 36568 | 428 | 15 | 13.7 |
| 6 | probable ABC-type transport system ATP-binding protein | ZP_02735300 | 31963 | 410 | 21 | 29.5 |
| 6 | hypothetical protein GobsU_12240 | ZP_02732569 | 33038 | 399 | 12 | 32 |
| 6 (2) | hypothetical protein GobsU_11080 | ZP_02732339 | 35572 | 357 | 28 | 18.9 |
| 6 (2) | hypothetical protein GobsU_36654 | ZP_02737399 | 34611 | 335 | 15 | 28.2 |
| 6 (2) | hypothetical protein GobsU_14991 | ZP_02733106 | 39437 | 691 | 30 | 30.5 |
| 6 (2,3) | hypothetical protein GobsU_26271 | ZP_02735340 | 33045 | 327 | 17 | 17.8 |
| 6 | oxidoreductase, short-chain dehydrogenase/reductase family protein | ZP_02734437 | 42703 | 368 | 20 | 19.2 |
| 6 | peptidylprolyl isomerase FKBP-type | ZP_02737067 | 31340 | 273 | 22 | 15 |
| 6 (3) | 50S ribosomal protein L4 | ZP_02734662 | 25590 | 263 | 7 | 20 |
| 6 | probable transport ATP-binding protein | ZP_02737283 | 33853 | 250 | 6 | 14.6 |
| 6 (2,3) | hypothetical protein GobsU_11730 | ZP_02732467 | 38243 | 243 | 22 | 16.7 |
| 6 | hypothetical protein GobsU_26666 | ZP_02735419 | 36271 | 404 | 16 | 21.2 |
| 6 | hypothetical protein GobsU_36085 | ZP_02737286 | 36120 | 186 | 3 | 12.9 |
| 6 | Translation elongation factor Ts (EF-Ts) | ZP_02737377 | 30314 | 180 | 8 | 14.8 |
| 6 | hypothetical protein GobsU_18952 | ZP_02733887 | 35360 | 238 | 25 | 16.6 |
| 6 | hypothetical protein GobsU_14996 | ZP_02733107 | 26205 | 160 | 4 | 17.1 |
| 6 (2,3) | hypothetical protein GobsU_24726 | ZP_02735033 | 29233 | 156 | 6 | 16.7 |
| 6 (2,3) | 30S ribosomal protein S3 | ZP_02734657 | 28381 | 143 | 8 | 12.5 |
| 6 (3) | hypothetical protein GobsU_20093 | ZP_02734114 | 28967 | 116 | 2 | 11.4 |
| 6 | ABC transporter, ATP-binding protein | ZP_02732799 | 35037 | 822 | 38 | 40.7 |
| 6 | ABC transporter, ATP-binding protein | ZP_02737368 | 35182 | 617 | 33 | 35.5 |
| 6 | ATP synthase gamma subunit | ZP_02731445 | 33052 | 1064 | 90 | 47.2 |
| 6 (2) | hypothetical protein GobsU_17530 | ZP_02733607 | 26219 | 634 | 95 | 51 |
| 6 | ABC transporter, ATPase subunit | ZP_02736275 | 34092 | 433 | 15 | 25.8 |
| 6 (3) | 50S ribosomal protein L3 | ZP_02737246 | 32224 | 615 | 30 | 32.5 |
| 6 (2) | hypothetical protein GobsU_14709 | ZP_02733050 | 37118 | 339 | 35 | 25.1 |
| 6 | hypothetical protein GobsU_35638 | ZP_02737197 | 34223 | 272 | 8 | 13.5 |
| 6 | probable protein kinase yloP | ZP_02730388 | 33059 | 474 | 15 | 34.6 |
| 6 (2) | hypothetical protein GobsU_32284 | ZP_02736536 | 34391 | 263 | 10 | 21.5 |
| 6 | putative serine/threonine-protein kinase | ZP_02735907 | 35707 | 249 | 10 | 13.7 |
| 6 | hypothetical protein GobsU_37737 | ZP_02737612 | 38778 | 223 | 6 | 10 |
| 6 | hypothetical protein GobsU_13797 | ZP_02732874 | 29652 | 214 | 10 | 15 |
| 6 | Squalene/phytoene synthase | ZP_02731127 | 34549 | 208 | 7 | 12.6 |
| 6 | Ribose transporter, periplasmic binding protein | ZP_02736325 | 35409 | 1030 | 73 | 51.2 |
| 6 (2) | F0F1 ATP synthase subunit beta | ZP_02731447 | 52132 | 1133 | 40 | 51.1 |
| 6 | hypothetical protein GobsU_05738 | ZP_02731277 | 31560 | 189 | 8 | 16.6 |
| 6 | hypothetical protein GobsU_19803 | ZP_02734056 | 30577 | 584 | 36 | 38.5 |
| 6 | periplasmic solute binding protein | ZP_02732461 | 34514 | 627 | 24 | 33.8 |
| 6 | ferrochelatase | ZP_02735861 | 36562 | 183 | 8 | 13.7 |
| 6 | succinic semialdehyde dehydrogenase | ZP_02736639 | 27647 | 235 | 6 | 20.3 |
| 6 (2) | cobalt-zinc-cadmium resistance protein | ZP_02733511 | 54820 | 512 | 14 | 24.3 |
| 6 | formate dehydrogenase beta subunit | ZP_02733206 | 35765 | 127 | 4 | 11 |
| 6 (2,3) | 30S ribosomal protein S2 | ZP_02737376 | 26492 | 319 | 14 | 32.9 |
| 6 | hypothetical protein GobsU_18957 | ZP_02733888 | 29436 | 106 | 4 | 10 |
| 6 | Alcohol dehydrogenase, zinc-binding domain protein | ZP_02737665 | 35836 | 749 | 23 | 42.6 |
| 6 | putative zinc ABC transporter, zinc-binding protein | ZP_02736154 | 36873 | 398 | 20 | 18.7 |
| 6 (2) | PfkB domain protein | ZP_02737066 | 39152 | 614 | 7 | 34.9 |
| 6 (2) | hypothetical protein GobsU_20238 | ZP_02734143 | 37132 | 461 | 4 | 29.2 |
| 6 (2,3) | hypothetical protein GobsU_12210 | ZP_02732563 | 41758 | 454 | 5 | 25.1 |
| 6 | Peptidase S1 and S6, chymotrypsin/Hap | ZP_02736370 | 44435 | 259 | 7 | 16.6 |
| 6 | MoxR-related ATPase, AAA superfamily protein | ZP_02732960 | 36904 | 250 | 7 | 16.7 |
| 6 (3) | hypothetical protein GobsU_01252 | ZP_02730395 | 38353 | 387 | 5 | 15.8 |
| 6 | phosphate ABC transporter, substrate-binding protein PstS | ZP_02733764 | 37171 | 347 | 2 | 20.1 |
| 6 (3) | flagellar motor switch protein G | ZP_02730986 | 32972 | 237 | 3 | 20.4 |
| 6 | UDP-glucose 4-epimerase | ZP_02736901 | 36518 | 209 | 6 | 12.6 |
| 6 | hypothetical protein GobsU_23012 | ZP_02734693 | 34897 | 208 | 6 | 11 |
| 6 | hypothetical protein GobsU_29321 | ZP_02735947 | 36283 | 193 | 8 | 10.7 |
| 6 | sulfate-binding protein precursor | ZP_02733577 | 37509 | 192 | 7 | 10 |
| 6 | ROK family protein | ZP_02735314 | 34351 | 181 | 2 | 9.9 |
| 6 | hypothetical protein GobsU_19329 | ZP_02733962 | 36927 | 180 | 2 | 11.1 |
| 6 | Alcohol dehydrogenase | ZP_02737751 | 35170 | 177 | 2 | 16.5 |
| 6 | hypothetical protein GobsU_39208 | ZP_02737905 | 37781 | 640 | 26 | 26.4 |
| 6 | type IV fimbrial assembly protein PilC | ZP_02734147 | 45854 | 591 | 28 | 24.4 |
| 6 | 2-hydroxyglutarate dehydrogenase | ZP_02730858 | 43266 | 157 | 7 | 12.1 |
| 6 | branched-chain amino acid transport ATP-binding protein | ZP_02731797 | 41111 | 172 | 6 | 8.8 |
| 6 (2,3) | hypothetical protein GobsU_20233 | ZP_02734142 | 48877 | 605 | 28 | 25.7 |
| 6 | hypothetical protein GobsU_34582 | ZP_02736992 | 44739 | 180 | 5 | 9.4 |
| 6 | ABC transporter related protein | ZP_02736413 | 36171 | 569 | 43 | 37.2 |
| 6 | glycosyl transferase, group 1 family protein | ZP_02730883 | 36479 | 331 | 6 | 16.8 |
| 6 | glycosyl transferase, group 1 family protein | ZP_02730879 | 40001 | 224 | 8 | 10.9 |
| 6 | glycosyl transferase, group 1 family protein | ZP_02730886 | 42261 | 167 | 3 | 10.3 |
| 6 | glycosyl transferase, group 1 | ZP_02730871 | 41119 | 626 | 22 | 38.8 |
| 6 | glycosyl transferase, group 1 | ZP_02730872 | 39488 | 280 | 10 | 21.5 |
| 6 | glycosyl transferase, group 1 | ZP_02731829 | 44120 | 311 | 7 | 18.4 |
| 6 | glycosyl transferase, group 2 family protein | ZP_02736170 | 34002 | 901 | 47 | 53.9 |
| 6 | glycosyl transferase, group 2 family protein | ZP_02735160 | 28363 | 440 | 12 | 31.5 |
| 6 | glycosyl transferase family 2 | ZP_02732222 | 32359 | 235 | 13 | 20.1 |
| 6 | glycosyl transferase family 2 | ZP_02730876 | 39841 | 131 | 5 | 13.4 |
| 6 (2) | Sulphate transport system permease protein 1 | ZP_02732762 | 39652 | 489 | 15 | 26.2 |
| 6 (2) | ketol-acid reductoisomerase | ZP_02737871 | 38760 | 350 | 10 | 18.6 |
| 6 (2,3) | putative peptidase | ZP_02736310 | 42703 | 734 | 30 | 35.4 |
| 6 (2) | hypothetical protein GobsU_20243 | ZP_02734144 | 41028 | 478 | 18 | 26.5 |
| 6 | HlyD family secretion protein, putative | ZP_02733129 | 37920 | 343 | 8 | 16.4 |
| 6 (2,3) | hypothetical protein GobsU_20643 | ZP_02734224 | 92712 | 750 | 25 | 19.2 |
| 6 | hypothetical protein GobsU_19334 | ZP_02733963 | 39693 | 299 | 10 | 17.9 |
| 6 (2) | Aldose 1-epimerase | ZP_02734020 | 41900 | 296 | 7 | 14.5 |
| 6 (2) | DNA-directed RNA polymerase subunit alpha | ZP_02733781 | 37755 | 728 | 39 | 25.6 |
| 6 | hypothetical protein GobsU_22142 | ZP_02734523 | 40583 | 278 | 12 | 12.1 |
| 6 | probable oxidoreductase | ZP_02734812 | 37679 | 219 | 8 | 15.4 |
| 6 | FMN-dependent alpha-hydroxy acid dehydrogenase | ZP_02735804 | 43337 | 554 | 18 | 32.2 |
| 6 | glyceraldehyde-3-phosphate dehydrogenase | ZP_02733850 | 37727 | 218 | 9 | 11.4 |
| 6 | 3-beta hydroxysteroid dehydrogenase/isomerase | ZP_02737474 | 35702 | 220 | 6 | 13.1 |
| 6 | hypothetical protein GobsU_14152 | ZP_02732939 | 44295 | 322 | 10 | 18.9 |
| 6 (2) | hypothetical protein GobsU_29901 | ZP_02736061 | 51041 | 527 | 18 | 20.1 |
| 6 (2) | hypothetical protein GobsU_04644 | ZP_02731061 | 47023 | 233 | 8 | 11.3 |
| 6 | probable Zn-dependent alcohol dehydrogenase | ZP_02734440 | 42880 | 372 | 17 | 19 |
| 6 | muconate cycloisomerase | ZP_02733879 | 43181 | 274 | 9 | 13.8 |
| 6 | succinyl-CoA synthetase (beta subunit) | ZP_02732131 | 41963 | 418 | 12 | 17.9 |
| 6 (2,3) | oxidoreductase domain protein | ZP_02735697 | 47995 | 777 | 36 | 32.8 |
| 6 | acriflavine resistance protein A | ZP_02731890 | 42775 | 1106 | 90 | 45 |
| 6 | transaldolase | ZP_02736346 | 38880 | 632 | 20 | 37.8 |
| 6 (2) | serine proteinase, HtrA/DegQ/DegS family | ZP_02735252 | 42708 | 216 | 5 | 10.9 |
| 6 (2) | hypothetical protein GobsU_26261 | ZP_02735338 | 45236 | 530 | 15 | 26.2 |
| 6 | amine oxidase, flavin-containing | ZP_02732954 | 43795 | 538 | 6 | 32.6 |
| 6 | xylose isomerase | ZP_02733161 | 49224 | 207 | 6 | 10.1 |
| 6 | hypothetical protein GobsU_33144 | ZP_02736706 | 44110 | 191 | 5 | 10.5 |
| 6 (2,3) | translation-associated GTPase | ZP_02730857 | 39663 | 193 | 6 | 10.4 |
| 6 | D-amino acid dehydrogenase, small chain | ZP_02737095 | 45047 | 192 | 7 | 9 |
| 6 | YcjX-like protein | ZP_02732078 | 51480 | 512 | 21 | 24.8 |
| 6 | secretion protein HlyD | ZP_02732105 | 50180 | 403 | 13 | 16.1 |
| 6 | hypothetical protein GobsU_13427 | ZP_02732802 | 37480 | 155 | 6 | 10.8 |
| 6 | oxidoreductase | ZP_02731486 | 44071 | 157 | 4 | 11.2 |
| 6 | hypothetical protein GobsU_31434 | ZP_02736366 | 50078 | 219 | 7 | 11.7 |
| 6 (2) | elongation factor Tu | ZP_02733080 | 47629 | 1210 | 14 | 55.1 |
| 6 | probable protein phosphatase 1 | ZP_02730410 | 47515 | 755 | 41 | 40.3 |
| 6 | efflux transporter, RND family, MFP subunit | ZP_02734533 | 48327 | 391 | 12 | 15.8 |
| 6 | efflux transporter, RND family, MFP subunit | ZP_02735371 | 43840 | 219 | 9 | 14 |
| 6 | NADH dehydrogenase subunit D | ZP_02732662 | 46011 | 549 | 7 | 24.4 |
| 6 | FAD-dependent pyridine nucleotide-disulphide oxidoreductase | ZP_02734627 | 47231 | 687 | 32 | 29.9 |
| 6 | dihydrolipoamide dehydrogenase | ZP_02731257 | 50105 | 406 | 13 | 23.4 |
| 6 | hypothetical protein GobsU_20203 | ZP_02734136 | 44521 | 450 | 13 | 26.1 |
| 6 | Enolase | ZP_02736401 | 46429 | 468 | 18 | 23.8 |
| 6 (2) | twitching mobility protein PilT | ZP_02734150 | 43895 | 507 | 16 | 30.2 |
| 6 | Catalase domain protein | ZP_02733351 | 39161 | 509 | 39 | 28.1 |
| 6 (2) | Cobalamin synthesis protein/P47K | ZP_02730707 | 41940 | 317 | 3 | 17.6 |
| 6 | hypothetical protein GobsU_33069 | ZP_02736691 | 88468 | 1546 | 88 | 43.8 |
| 6 (2,3) | hypothetical protein GobsU_29906 | ZP_02736062 | 76943 | 1018 | 33 | 31.5 |
| 6 | cell division protein FtsH | ZP_02737094 | 76041 | 502 | 24 | 20.1 |
| 6 | dihydrolipoamide acetyltransferase | ZP_02733334 | 43017 | 414 | 15 | 20.4 |
| 6 (2) | pyruvate dehydrogenase complex, dihydrolipoamide acetyltransferase E2 component | ZP_02735609 | 56948 | 642 | 21 | 21.2 |
| 6 (2) | twitching motility protein PilT | ZP_02733546 | 43722 | 391 | 16 | 27.8 |
| 6 (2) | 6-phosphogluconate dehydrogenase | ZP_02734911 | 52647 | 392 | 12 | 17.3 |
| 6 | acyl-CoA dehydrogenase domain protein | ZP_02734570 | 41522 | 273 | 9 | 12.8 |
| 6 | phosphoglycerate kinase | ZP_02733849 | 42098 | 255 | 7 | 11.9 |
| 6 | probable tetraacyldisaccharide 4-kinase | ZP_02736188 | 36745 | 242 | 7 | 18.2 |
| 6 | hypothetical protein GobsU_07767 | ZP_02731678 | 52109 | 754 | 29 | 29.4 |
| 6 | hypothetical protein GobsU_29508 | ZP_02735984 | 53183 | 657 | 30 | 31.2 |
| 6 (2) | hypothetical protein GobsU_23532 | ZP_02734797 | 61448 | 1390 | 66 | 37.7 |
| 6 | type I phosphodiesterase/nucleotide pyrophosphatase | ZP_02737031 | 50667 | 567 | 19 | 25.9 |
| 6 | putative auxin-regulated protein | ZP_02734402 | 58942 | 963 | 35 | 34 |
| 6 (2) | probable auxin-responsive-like protein | ZP_02733619 | 63167 | 158 | 4 | 6.9 |
| 6 | proton-dependent oligopeptide transporter family protein | ZP_02734743 | 64966 | 266 | 9 | 13.4 |
| 6 | C-terminal processing peptidase S41A | ZP_02737571 | 25174 | 268 | 8 | 13.4 |
| 6 | hypothetical protein GobsU_20208 | ZP_02734137 | 52065 | 316 | 21 | 16 |
| 6 (2,3) | Flagellar FliF M-ring protein | ZP_02730987 | 54974 | 693 | 26 | 26 |
| 6 | hypothetical protein GobsU_01882 | ZP_02730517 | 49589 | 253 | 15 | 10 |
| 6 (2) | Ferredoxin | ZP_02732656 | 63129 | 1306 | 71 | 44.7 |
| 6 (2,3) | hypothetical protein GobsU_04904 | ZP_02731113 | 61669 | 596 | 19 | 20.8 |
| 6 | hypothetical protein GobsU_23022 | ZP_02734695 | 57316 | 392 | 11 | 15.6 |
| 6 (2) | sigma-54 dependent transcriptional regulator/response regulator | ZP_02732401 | 53500 | 572 | 14 | 23.2 |
| 6 | FAD dependent oxidoreductase | ZP_02735201 | 58357 | 531 | 17 | 25.3 |
| 6 (2) | RNA binding S1 domain protein | ZP_02737093 | 118263 | 500 | 18 | 11.2 |
| 6 | Acyl-CoA dehydrogenase | ZP_02732342 | 65323 | 1571 | 120 | 39 |
| 6 (2) | sialic acid-specific 9-O-acetylesterase | ZP_02737096 | 57483 | 497 | 14 | 15.9 |
| 6 (2) | hypothetical protein GobsU_34522 | ZP_02736980 | 59238 | 443 | 12 | 16.2 |
| 6 (2) | hypothetical protein GobsU_14549 | ZP_02733018 | 54836 | 443 | 12 | 20.2 |
| 6 | hypothetical protein GobsU_06520 | ZP_02731433 | 50596 | 338 | 12 | 13.6 |
| 6 (2,3) | hypothetical protein GobsU_38147 | ZP_02737694 | 46334 | 580 | 36 | 23.1 |
| 6 (2) | WD-40 repeat | ZP_02737050 | 72113 | 870 | 47 | 22.3 |
| 6 (2) | hypothetical protein GobsU_16609 | ZP_02733426 | 56115 | 758 | 54 | 27.3 |
| 6 | hypothetical protein GobsU_25556 | ZP_02735199 | 62055 | 691 | 36 | 20.8 |
| 6 | Phytoene dehydrogenase and related protein-like protein | ZP_02736586 | 50255 | 207 | 5 | 7.4 |
| 6 | hypothetical protein GobsU_28340 | ZP_02735752 | 46838 | 296 | 10 | 16 |
| 6 | probable PbrT protein-possibly cytochrome c | ZP_02733592 | 52631 | 312 | 10 | 12.1 |
| 6 (2) | probable DNA-directed RNA polymerase alpha chain | ZP_02736955 | 51701 | 322 | 7 | 10.7 |
| 6 (2) | Signal Transduction Histidine Kinases (STHK) | ZP_02734429 | 58312 | 495 | 13 | 16.4 |
| 6 (2) | fumarate hydratase | ZP_02735455 | 54808 | 438 | 17 | 19.8 |
| 6 (2) | succinate dehydrogenase flavoprotein subunit | ZP_02737002 | 70751 | 1198 | 54 | 32.2 |
| 6 (2) | heat shock protein GroEL | ZP_02737557 | 58429 | 348 | 11 | 17.4 |
| 6 (2) | probable chemotaxis transducer | ZP_02735177 | 59822 | 318 | 7 | 16.7 |
| 6 (2) | hypothetical protein GobsU_24941 | ZP_02735076 | 56127 | 299 | 10 | 13.8 |
| 6 | probable protein kinase yloP-putative serine/threonine protein kinase | ZP_02733729 | 56940 | 247 | 9 | 15.4 |
| 6 (2) | hypothetical protein GobsU_27341 | ZP_02735553 | 61963 | 212 | 7 | 8.3 |
| 6 | hypothetical protein GobsU_31909 | ZP_02736461 | 59664 | 209 | 8 | 12 |
| 6 (2,3) | hypothetical protein GobsU_38668 | ZP_02737797 | 58509 | 382 | 16 | 13.9 |
| 6 (2) | 30S ribosomal protein S1 | ZP_02730364 | 57995 | 218 | 8 | 6.8 |
| 6 (2,3) | 30S ribosomal protein S1 | ZP_02736868 | 69112 | 803 | 38 | 28.6 |
| 6 (2) | heat shock protein 70 family protein | ZP_02735562 | 66391 | 798 | 26 | 31.6 |
| 6 (2) | 2-isopropylmalate synthase | ZP_02732952 | 57498 | 188 | 7 | 10.4 |
| 6 (2) | PrkA AAA domain protein | ZP_02733099 | 79001 | 1058 | 31 | 29.7 |
| 6 (2) | hypothetical protein GobsU_19384 | ZP_02733973 | 47872 | 849 | 37 | 31.5 |
| 6 (2) | hypothetical protein GobsU_20458 | ZP_02734187 | 63184 | 674 | 21 | 23.8 |
| 6 (2) | flagellin FliC | ZP_02737314 | 61923 | 1272 | 59 | 40.6 |
| 6 | delta-1-pyrroline-5-carboxylate dehydrogenase | ZP_02735812 | 112374 | 1133 | 35 | 23.3 |
| 6 | peptidase S45 penicillin amidase | ZP_02735855 | 85148 | 348 | 11 | 9.6 |
| 6 | probable NADH-dependent dehydrogenase | ZP_02731432 | 63505 | 917 | 37 | 28.8 |
| 6 | 60 kDa chaperonin 5 | ZP_02736489 | 60169 | 501 | 22 | 20.3 |
| 6 (2) | type IV fimbrial assembly protein PilB | ZP_02734151 | 63301 | 917 | 37 | 28.8 |
| 6 (2) | trigger factor | ZP_02733942 | 55785 | 443 | 14 | 16.3 |
| 6 | hypothetical protein GobsU_23972 | ZP_02734885 | 20528 | 245 | 7 | 30.8 |
| 6 (2) | hypothetical protein GobsU_21120 | ZP_02734319 | 66262 | 192 | 6 | 8.1 |
| 6 (2) | alkaline phosphatase | ZP_02732407 | 66257 | 1360 | 50 | 44.5 |
| 6 (2) | 60 kDa chaperonin | ZP_02736491 | 61375 | 250 | 8 | 10 |
| 6 (2) | hypothetical protein GobsU_30770 | ZP_02736234 | 68086 | 685 | 21 | 22.7 |
| 6 (2) | glycyl-tRNA synthetase | ZP_02736496 | 63211 | 333 | 11 | 16.2 |
| 6 (2,3) | glutamine synthetase, catalytic region | ZP_02737578 | 79979 | 1721 | 88 | 43.2 |
| 6 | NADH dehydrogenase (quinone) | ZP_02734324 | 76409 | 1020 | 41 | 25.5 |
| 6 | probable signal peptidase I | ZP_02732748 | 68998 | 629 | 24 | 18.6 |
| 6 (2) | threonyl-tRNA synthetase | ZP_02734724 | 70497 | 461 | 12 | 14.7 |
| 6 | hypothetical protein GobsU_08402 | ZP_02731805 | 68867 | 369 | 8 | 13.7 |
| 6 (2) | hypothetical protein GobsU_30765 | ZP_02736233 | 70604 | 580 | 23 | 16 |
| 6 (2) | GTP-binding elongation factor | ZP_02732217 | 67411 | 192 | 6 | 7.7 |
| 6 (2,3) | hypothetical protein GobsU_29154 | ZP_02735914 | 33558 | 158 | 4 | 8.6 |
| 6 (3) | 50S ribosomal protein L5 | ZP_02734651 | 20975 | 133 | 3 | 13.1 |
| 6 (3) | Scramblase family protein | ZP_02730790 | 21656 | 388 | 6 | 39.4 |
| 6 (2,3) | hypothetical protein GobsU_20648 | ZP_02734225 | 74282 | 940 | 29 | 23.7 |
| 6 | hypothetical protein GobsU_38418 | ZP_02737747 | 14344 | 312 | 22 | 39 |
| 6 | probable ribosomal protein S6 | ZP_02736394 | 20250 | 107 | 2 | 15.4 |
| 6 | probable 30S ribosomal protein S17 | ZP_02734654 | 13764 | 145 | 9 | 16.3 |
| 6 | hypothetical protein GobsU_33074 | ZP_02736692 | 89212 | 299 | 7 | 9.1 |
| 6 (2) | methylcitrate synthase | ZP_02732426 | 42530 | 164 | 6 | 9.2 |
| 6 | probable ATP synthase CF1 subunit e | ZP_02731448 | 14248 | 446 | 37 | 61.2 |
| 6 (2) | hypothetical protein GobsU_30964 | ZP_02736272 | 23254 | 382 | 9 | 30.4 |
| 6 | hypothetical protein GobsU_11645 | ZP_02732450 | 14806 | 261 | 12 | 41.3 |
| 6 | hypothetical protein GobsU_36060 | ZP_02737281 | 15530 | 93 | 3 | 12.2 |
| 6 | hypothetical protein GobsU_21075 | ZP_02734310 | 16557 | 248 | 10 | 38.3 |
| 6 (3) | hypothetical protein GobsU_14884 | ZP_02733085 | 19370 | 236 | 7 | 19.7 |
| 6 | hypothetical protein GobsU_35800 | ZP_02737229 | 13495 | 233 | 12 | 18 |
| 6 (2) | hypothetical protein GobsU_11415 | ZP_02732406 | 14995 | 220 | 30 | 27.5 |
| 6 | hypothetical protein GobsU_05084 | ZP_02731147 | 15541 | 209 | 10 | 24.3 |
| 6 | hypothetical protein GobsU_26206 | ZP_02735327 | 17561 | 161 | 5 | 20.3 |
| 6 | HflC protein | ZP_02732735 | 37784 | 152 | 6 | 8.5 |
| 6 | hypothetical protein GobsU_27231 | ZP_02735531 | 12326 | 150 | 2 | 14.8 |
| 6 | hypothetical protein GobsU_36340 | ZP_02737337 | 14636 | 149 | 4 | 25.5 |
| 6 | probable anti-anti-sigma regulatory factor (antagonist of anti-sigma factor) | ZP_02734268 | 13732 | 146 | 14 | 25.6 |
| 6 | hypothetical protein GobsU_29040 | ZP_02735892 | 10640 | 112 | 4 | 26.3 |
| 6 (2) | hypothetical protein GobsU_17456 | ZP_02733593 | 12531 | 103 | 4 | 24.6 |
| 6 | riboflavin synthase subunit beta | ZP_02732766 | 16302 | 171 | 6 | 20.8 |
| 6 | hypothetical protein GobsU_22822 | ZP_02734655 | 16991 | 101 | 7 | 17.4 |
| 6 | hypothetical protein GobsU_30195 | ZP_02736119 | 10341 | 99 | 2 | 20.4 |
| 6 | hypothetical protein GobsU_14469 | ZP_02733002 | 11672 | 91 | 3 | 13.9 |
| 6 (2) | multidrug efflux system, HlyD family subunit | ZP_02734259 | 47621 | 476 | 29 | 18 |
| 6 (2) | efflux transporter, RND family, MFP subunit | ZP_02732378 | 48939 | 302 | 19 | 11 |
| 6 | hypothetical protein GobsU_21140 | ZP_02734323 | 59330 | 216 | 6 | 6.4 |
| 6 | hypothetical protein GobsU_18305 | ZP_02733762 | 57398 | 143 | 8 | 9.3 |
| 6 | Molybdopterin oxidoreductase, iron-sulfur binding subunit | ZP_02736517 | 125542 | 1687 | 64 | 32.1 |
| 6 (2) | hypothetical protein GobsU_34912 | ZP_02737058 | 127649 | 1168 | 35 | 20.6 |
| 6 | GAF sensor hybrid histidine kinase | ZP_02735634 | 125815 | 478 | 12 | 8 |
| 6 | transporter, hydrophobe/amphiphile efflux-1 (HAE1) family protein | ZP_02732379 | 116833 | 337 | 10 | 5.5 |
| 6 | hypothetical protein GobsU_12857 | ZP_02732690 | 106371 | 495 | 11 | 10.3 |
| 6 | cation efflux system protein CZCA | ZP_02733510 | 111095 | 299 | 10 | 6.1 |
| 6 | DNA gyrase subunit A | ZP_02737492 | 96932 | 311 | 10 | 7.2 |
| 6 (2,3) | hypothetical protein GobsU_20253 | ZP_02734146 | 26677 | 426 | 14 | 39.1 |
| 6 (2,3) | 30S ribosomal protein S4 | ZP_02736977 | 22489 | 379 | 15 | 23.5 |
| 6 (2) | ABC transporter (glutamine transport ATP-binding protein) | ZP_02735279 | 25792 | 180 | 6 | 21.7 |
| 6 (2) | hypothetical protein GobsU_26266 | ZP_02735339 | 27891 | 161 | 8 | 13.3 |
| 6 (2,3) | hypothetical protein GobsU_25221 | ZP_02735132 | 31969 | 150 | 4 | 12.3 |
| 6 (2) | chaperone protein HtpG | ZP_02733601 | 27919 | 138 | 9 | 11.8 |
| 6 (2) | lipoprotein releasing system ATP-binding protein lolD | ZP_02734712 | 24120 | 94 | 3 | 12.5 |
| 6 | putative serine protease containing two PDZ domains | ZP_02734485 | 31120 | 116 | 3 | 7.3 |
| **Summary for Fraction 6** | **184 proteins are unique for this fraction (in green) – 57.7% of total** | **34 proteins overlap with proteins from fractions 2 and 3** | **82 proteins overlap with proteins from fraction 2** | **19 proteins overlap with proteins from fraction 3** |  | **319 proteins in total** |

* as calculated by MASCOT using WAL-1 draft genome project. NCBI data may differ from those given by WAL-1.
