# Supplementary material for: Nuclear Pore-Like Structures in a Compartmentalized Bacterium
Source: PLoS One. 2017 Feb 1;12(2):e0169432. doi: 10.1371/journal.pone.0169432 (PMC5287468; doi:10.1371/journal.pone.0169432)
Supplement: S2 Table — (DOC) [file pone.0169432.s025.doc]

**S2 Table. Summary of the membrane proteome analysis.**

**
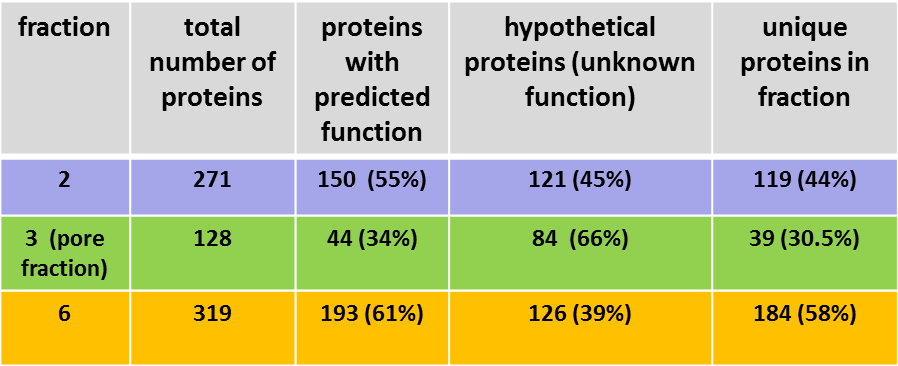
**

The proteins identified by mass-spectrometry are grouped according to the NCBI annotation as hypothetical proteins with no predicted function or with firmly predicted function (such as ribosomal proteins, ABC transporters etc).
