# Supplementary material for: Nuclear Pore-Like Structures in a Compartmentalized Bacterium
Source: PLoS One. 2017 Feb 1;12(2):e0169432. doi: 10.1371/journal.pone.0169432 (PMC5287468; doi:10.1371/journal.pone.0169432)
Supplement: S4 Table — (DOC) [file pone.0169432.s027.doc]

**S4 Table. Results from structural analysis for the C-terminal region of cluster 1 (β-propeller) protein constituents***

| ID | Fraction | Confidence | Coverage | PDB template |
| --- | --- | --- | --- | --- |
| ZP_02731030 | (3) | 16.0% | 1% | 3IKM |
| ZP_02731113 | (2,3,6) | 99.9 | 52% | 2C4D |
| ZP_02733245 | (2,3) | 99.9% | 42% | 2C4D |
| ZP_02734577 | (3) | 98.6% | 25% | 2C4D |
| ZP_02734776 | (3) | 99.9% | 25% | 2C4D |
| ZP_02734818 | (3) | 40.1% | 5% | 3FCS |
| ZP_02735782 | (3) | 1.6% | 9% | 3RB7 |
| ZP_02736670 | (3) | 100% | 76% | 2C4D |
| ZP_02737072 | (3) | 99.8 | 39% | 2C4D |
| ZP_02737073 | (2,3) | 100% | 91% | 2C4D |
| ZP_02737797 | (2,3,6) | 99.9% | 49% | 2C4D |

*Models generated from full sequences except ZP_02734818 (see text). Best hits (shown) were chosen based on combined top confidence and coverage scores, and location in the alignable C-terminus (Figure supplement 13). Column 1, Genbank accessions. Columns 2 and 3, confidence and coverage scores as generated by Phyre2. Column 4, PDB template ID used by Phyre2 to generate models. Results below 95% confidence were not further analysed.
