# Supplementary material for: Nuclear Pore-Like Structures in a Compartmentalized Bacterium
Source: PLoS One. 2017 Feb 1;12(2):e0169432. doi: 10.1371/journal.pone.0169432 (PMC5287468; doi:10.1371/journal.pone.0169432)
Supplement: S5 Table — (DOC) [file pone.0169432.s028.doc]

**S5 Table**. **Results from structural analysis of cluster 2 (pili) protein constituents***

| ID | Fraction | Confidence | Coverage | PDB template |
| --- | --- | --- | --- | --- |
| ZP_02731198 | (2,3) | 99.7% | 21% | 1OQW |
| ZP_02731806 | (2,3,6) | 99.6% | 23% | 1OQW |
| ZP_02732451 | (2,3) | 99.7% | 24% | 1OQW |
| ZP_02732467 | (2,3,6) | 99.7% | 23% | 1OQW |
| ZP_02733038 | (2,3) | 99.6% | 25% | 1OQW |
| ZP_02733041 | (2,3,6) | 99.7% | 27% | 1OQW |
| ZP_02735033 | (2,3,6) | 54.8% | 8% | 2PIL |
| ZP_02735132 | (2,3,6) | 99.7% | 27% | 1OQW |
| ZP_02735532 | (2,3,6) | 99.6% | 20% | 1OQW |
| ZP_02735914 | (2,3,6) | 99.7% | 26% | 1OQW |
| ZP_02737610 | (2,3) | 99.7% | 26% | 1OQW |

*Models generated from full sequences. Best hits (shown) were chosen based on combined top confidence and coverage scores. Column 1, Genbank accessions. Columns 2 and 3, confidence and coverage scores as generated by Phyre2. Column 4, PDB template ID used by Phyre2 to generate models.
