# Supplementary material for: Nuclear Pore-Like Structures in a Compartmentalized Bacterium
Source: PLoS One. 2017 Feb 1;12(2):e0169432. doi: 10.1371/journal.pone.0169432 (PMC5287468; doi:10.1371/journal.pone.0169432)
Supplement: S6 Table — (DOC) [file pone.0169432.s029.doc]

**S6 Table: Results from keyword analysis of Phyre output.**

ID Fraction Confidence Coverage PDB template

| | **outer membrane lipoprotein** | | | | |  | | --- | --- | --- | --- | --- | --- | | ID | Fraction | Confidence | Coverage | PDB template |  | | ZP_02730892 | (3) | 100.00% | 95.00% | 2J58 |  | | ZP_02731226 | (3) | 77.00% | 29.00% | 1JCC |  | | **ZP_02733516** | (3) | 100.00% | 75.00% | 2J58 |  | | **ZP_02736601** | (2,3) | 100.00% | 70.00% | 2J58 |  | |  |  |  |  |  |  | | **outer membrane efflux protein** | | | | |  | | ID | Fraction | Confidence | Coverage | PDB template |  | | ZP_02730507 | (3) | 99.00% | 67.00% | 1WP1 |  | | ZP_02731891 | (3) | 100.00% | 91.00% | 1WP1 |  | | ZP_02732067 | (3) | 99.70% | 91.00% | 1WP1 |  | | ZP_02732104 | (2,3) | 100.00% | 95.00% | 1WP1 |  | | **ZP_02732829** | (3) | 100.00% | 88.00% | 1WP1 |  | | **ZP_02735955** | (2,3) | 100.00% | 62.00% | 1WP1 |  | | ZP_02736193 | (3) | 100.00% | 89.00% | 1WP1 |  | |  |  |  |  |  |  | | **transmembrane beta barrel** | | | | |  | | ID | Fraction | Confidence | Coverage | PDB template |  | | ZP_02730574 | (3) | 99.90% | 58.00% | 3RBH |  | | **ZP_02731192** | (3) | 98.30% | 53.00% | 1I78 |  | | ZP_02732104 | (2,3) | 5.30% | 9.00% | 1QJ8 |  | | ZP_02732631 | (3) | 99.40% | 78.00% | 2O4V |  | | ZP_02734397 | (3) | 96.80% | 52.00% | 1I78 |  | | **ZP_02734840** | (2,3) | 97.40% | 46.00% | 1I78 |  | | ZP_02735776 | (3) | 97.90% | 60.00% | 2X27 |  | | ZP_02735845 | (2,3) | 100.00% | 64.00% | 3RBH |  | | ZP_02735880 | (3) | 97.60% | 11.00% | 1T16 |  | | ZP_02735955 | (2,3) | 38.80% | 6.00% | 2MPR |  | |  |  |  |  |  |  | | **porin** | | | | |  | | ID | Fraction | Confidence | Coverage | PDB template |  | | ZP_02730574 | (3) | 98.60% | 64.00% | 3SYB |  | | **ZP_02731192** | (3) | 20.80% | 9.00% | 2O4V |  | | ZP_02732631 | (3) | 99.40% | 78.00% | 2O4V |  | | ZP_02734397 | (3) | 36.80% | 61.00% | 1T16 |  | | **ZP_02734840** | (2,3) | 50.60% | 58.00% | 1T16 |  | | ZP_02735776 | (3) | 76.20% | 34.00% | 2WJQ |  | | ZP_02735845 | (2,3) | 100.00% | 64.00% | 2Y0K |  | | ZP_02735880 | (3) | 97.60% | 11.00% | 1T16 |  | | ZP_02735955 | (2,3) | 38.80% | 6.00% | 2MPR |  | |  |  |  |  |  |  | | **tolc** | | | | |  | | ID | Fraction | Confidence | Coverage | PDB template |  | | ZP_02730507 | (3) | 99.90% | 61.00% | 1TQQ |  | | ZP_02731891 | (3) | 100.00% | 89.00% | 1TQQ |  | | ZP_02732067 | (3) | 98.40% | 82.00% | 1TQQ |  | | ZP_02732104 | (2,3) | 100.00% | 99.00% | 1TQQ |  | | **ZP_02732829** | (3) | 100.00% | 80.00% | 1TQQ |  | | **ZP_02735955** | (2,3) | 100.00% | 60.00% | 1TQQ |  | | ZP_02736193 | (3) | 100.00% | 77.00% | 1TQQ |  | |  |  |  |  |  |  | | **oprX** | | | | | | | ID | Fraction | Confidence | Coverage | PDB template | Class | | ZP_02730574 | (3) | 97.10% | 61.00% | 2ODJ | oprd | | **ZP_02731192** | (3) | 96.50% | 48.00% | 2X27 | oprg | | ZP_02732631 | (3) | 99.40% | 78.00% | 2O4V | oprp | | ZP_02734397 | (3) | 91.00% | 40.00% | 2X27 | oprg | | **ZP_02734840** | (2,3) | 58.20% | 39.00% | 2LHF | oprh | | ZP_02735776 | (3) | 97.90% | 60.00% | 2X27 | oprg | | ZP_02735845 | (2,3) | 99.90% | 64.00% | 2ODJ | oprd | | ZP_02735880 | (3) | 78.90% | 5.00% | 2X27 | oprg | | ZP_02737902 | (2,3) | 5.60% | 10.00% | 2LHF | oprh | |  |  |  |  |  |  |   All the structural models generated by Phyre for the 128 fraction 3 proteins were screened for hits to bacterial transmembrane proteins using the keywords showed. A protein can have hits to more than one of these transmembrane protein classes. For each protein, we list the ID, the membrane fraction containing the protein, the confidence as calculated by Phyre2, the coverage of the sequence, and the PDB template used in the model. For the oprX proteins we also list the specific class. Bold entries show proteins that were clustered together as a duo in the clustering analysis. |  |
| --- | --- | --- | --- | --- | --- | --- | --- | --- | --- | --- | --- | --- | --- | --- | --- | --- | --- | --- | --- | --- | --- | --- | --- | --- | --- | --- | --- | --- | --- | --- | --- | --- | --- | --- | --- | --- | --- | --- | --- | --- | --- | --- | --- | --- | --- | --- | --- | --- | --- | --- | --- | --- | --- | --- | --- | --- | --- | --- | --- | --- | --- | --- | --- | --- | --- | --- | --- | --- | --- | --- | --- | --- | --- | --- | --- | --- | --- | --- | --- | --- | --- | --- | --- | --- | --- | --- | --- | --- | --- | --- | --- | --- | --- | --- | --- | --- | --- | --- | --- | --- | --- | --- | --- | --- | --- | --- | --- | --- | --- | --- | --- | --- | --- | --- | --- | --- | --- | --- | --- | --- | --- | --- | --- | --- | --- | --- | --- | --- | --- | --- | --- | --- | --- | --- | --- | --- | --- | --- | --- | --- | --- | --- | --- | --- | --- | --- | --- | --- | --- | --- | --- | --- | --- | --- | --- | --- | --- | --- | --- | --- | --- | --- | --- | --- | --- | --- | --- | --- | --- | --- | --- | --- | --- | --- | --- | --- | --- | --- | --- | --- | --- | --- | --- | --- | --- | --- | --- | --- | --- | --- | --- | --- | --- | --- | --- | --- | --- | --- | --- | --- | --- | --- | --- | --- | --- | --- | --- | --- | --- | --- | --- | --- | --- | --- | --- | --- | --- | --- | --- | --- | --- | --- | --- | --- | --- | --- | --- | --- | --- | --- | --- | --- | --- | --- | --- | --- | --- | --- | --- | --- | --- | --- | --- | --- | --- | --- | --- | --- | --- | --- | --- | --- | --- | --- | --- | --- | --- | --- | --- | --- | --- | --- | --- | --- | --- | --- | --- | --- | --- | --- | --- | --- | --- | --- | --- | --- | --- | --- | --- | --- | --- | --- | --- | --- | --- | --- | --- | --- | --- | --- | --- | --- | --- | --- | --- | --- | --- | --- | --- | --- | --- | --- | --- | --- | --- | --- | --- | --- | --- | --- | --- | --- | --- | --- | --- | --- | --- | --- | --- | --- | --- | --- | --- | --- | --- | --- | --- | --- | --- | --- | --- | --- | --- | --- | --- | --- | --- | --- | --- | --- | --- | --- | --- | --- | --- | --- | --- | --- | --- | --- | --- | --- | --- | --- | --- | --- | --- | --- | --- | --- | --- | --- | --- | --- | --- | --- | --- | --- | --- | --- | --- | --- | --- | --- | --- | --- | --- | --- | --- | --- | --- | --- | --- | --- | --- |
